# Fluorinated pore walls boost PFAS capture in mechanochemically synthesized covalent organic frameworks

**DOI:** 10.1039/d6ra06859f

**Published:** 2026-10-09

**Authors:** Maroof Arshadul Hoque, Tanja Westphalen, Stephanie Kluge, Christian Piechotta, Jana Falkenhagen, Nader de Sousa Amadeu, Carsten Prinz, Prasenjit Das, Ernesto Scoppola, Tomasz M. Stawski, Biswajit Bhattacharya, Franziska Emmerling

**Affiliations:** a BAM Federal Institute for Materials Research and Testing Richard-Willstätter-Str. 11 12489 Berlin Germany franziska.emmerling@bam.de biswajit.bhattacharya@bam.de; b Department of Chemistry, Humboldt University Berlin Brook-Taylor-Straße 2 12489 Berlin Germany; c Department of Chemistry, Functional Materials, Indian Institute of Technology Ropar SSB Block Rupnagar Punjab 140001 India; d Department of Biomaterials, Max Planck Institute of Colloids and Interfaces Am Mühlenberg 1 14476 Potsdam Germany

## Abstract

Per- and polyfluoroalkyl substances (PFAS) are persistent water contaminants that require robust, high-affinity sorbents. Covalent organic frameworks (COFs) provide chemically stable, modular platforms for capturing PFAS and mechanochemical synthesis offers an environmentally friendly, scalable method of production. In this study, we use mechanochemistry to quickly produce an isoreticular series of large-pore imine COFs (TAPB-TA, TAPB-DFTA and TAPB-DMTA) with systematically varied pore-wall functionality, which is optimised for PFAS adsorption. The fluorinated TAPB-DFTA COF exhibits the greatest crystallinity, the highest BET surface area (1237 m^2^ g^−1^) and the greatest thermal stability. These properties result in superior uptake of PFOA, PFOS and short-chain analogues compared to the methylated and unsubstituted analogues in batch adsorption experiments across a broad concentration range. The three COFs achieve near-quantitative PFOS removal within minutes, while TAPB-DFTA maintains significantly higher PFOA removal even at a feed concentration of 100 mg L^−1^. These findings indicate that mechanochemical synthesis combined with pore wall modification offers a possible approach for tuning COF adsorption sites for PFAS uptake.

## Abstract 2

Combining mechanochemical synthesis with pore wall modification provides a simple and sustainable way to tune COF adsorption sites for rapid and efficient PFAS removal from water.

## Graphical abstract

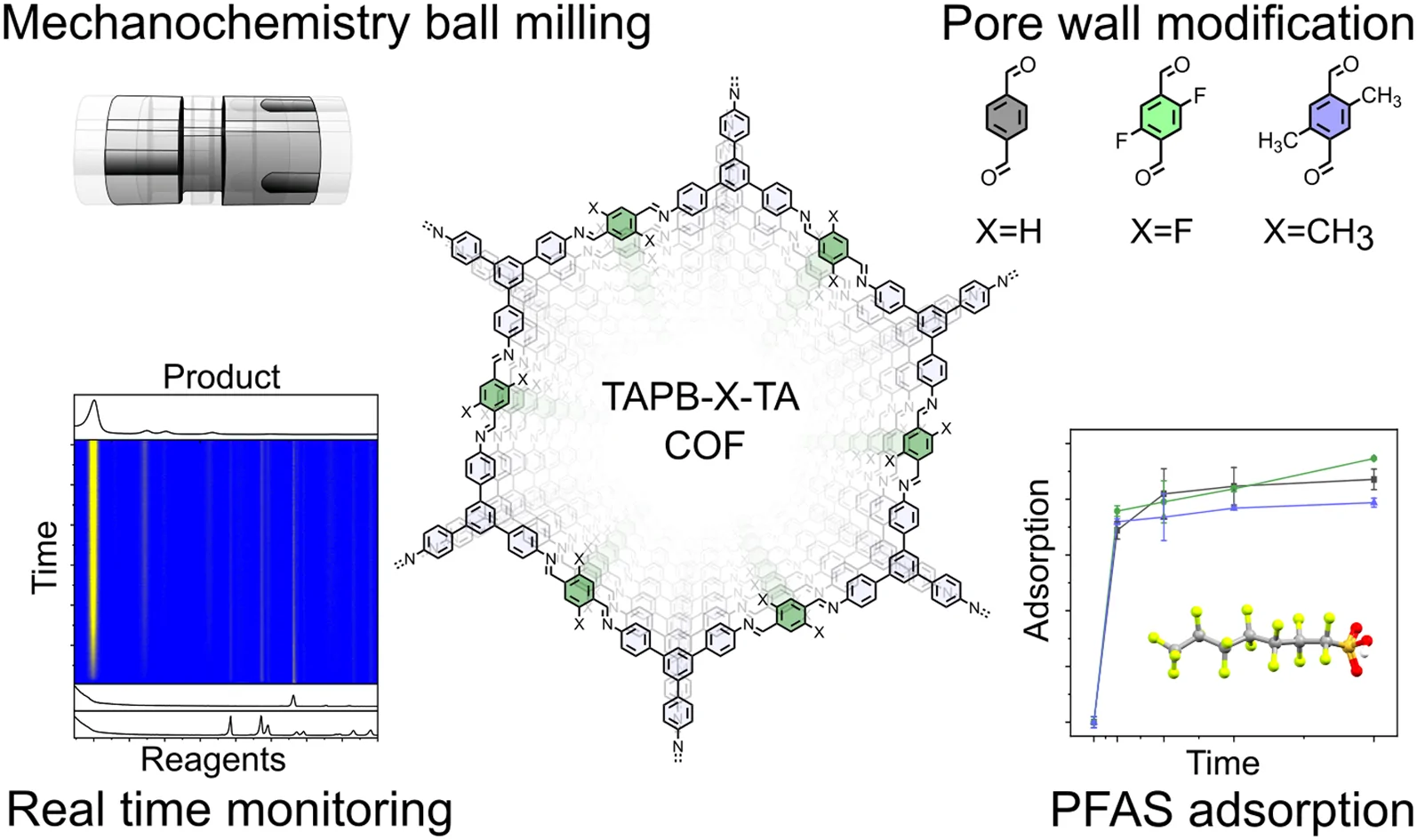

## Introduction

Per- and polyfluoroalkyl substances (PFAS) are persistent environmental contaminants known for their toxicity and bioaccumulation.^1,2^ They also exhibit extreme resistance to natural degradation processes.^3,4^ After decades of large scale production, PFAS have become ever present, infiltrating groundwater and even drinking water supplies.^5–7^ Detectable levels reported even in commercially bottled water have raised significant public concern.^6,8–10^ As PFAS exposure has been linked to carcinogenic outcomes, the presence of PFAS in drinking water poses a serious risk to human health and the environment.^11–13^

Conventional treatment methods to remove PFAS like activated carbon^14,15^ and ion-exchange resins^16,17^ often fall short. To address these limitations, recent efforts have focused on advanced porous materials, especially metal organic frameworks (MOFs) and covalent organic frameworks (COFs).^18^ MOFs have attracted significant attention because of their high surface areas, well-defined pore architectures and rich structural diversity tailored for host–guest interactions.^19–22^ However, many MOFs suffer from hydrolytic and chemical instability in aqueous environments.^23^ This can lead to partial framework degradation and potential metal leaching which can complicate long term PFAS adsorption application.^24^ In contrast, COFs combine similarly high porosity and tunable pore geometries with robust covalent bonding and customizable organic functional groups.^25–27^ They provide a more chemically stable foundation for improving PFAS affinity.^28^ Wang *et al.*^29^ demonstrated that COFs with pore sizes 2.5–4 times the length of the target PFAS maximize uptake by promoting molecular threading and reducing diffusion barriers. Zarei *et al.*^30^ reported that viologen based COFs achieve record adsorption capacities up to 2500 mg g^−1^, driven by electrostatic and dipolar interactions. They also observed the removal of various anionic PFAS at levels of 90–99%, where equilibrium was reached within 30 minutes. Zarei *et al.*^31^ also showed that olefin-linked COFs that feature hydrophobic surfaces and *ortho*-methoxy functionalities, reduce PFOA and PFOS from 20 µg L^−1^ to below EPA advisory limits (0.070 µg L^−1^). They also demonstrated that their materials maintain rapid kinetics, high efficiency and reusability.

Despite their potential, the real-world adoption of COFs is limited by the fact that solvothermal synthesis is energy-intensive and environmentally unfriendly.^32^ This synthesis method typically involves multiple freeze–thaw–vacuum cycles, the use of toxic solvents and elevated reaction temperatures. In addition, the reactions often require several days to reach completion.^33^ As an alternative, mechanochemistry has emerged as a more environmentally benign synthetic route, enabling solvent-free or minimal-solvent reactions under mild conditions that are driven by mechanical forces such as grinding and shearing.^34–42^ This synthesis route strongly aligns with the principles of green chemistry by eliminating the need for bulk solvents, improving energy efficiency and increasing overall process safety.^43–45^ Besides that, mechanochemistry offers a valuable opportunity to study reaction mechanisms through state-of-the-art time resolved *in situ* (TRIS) X-ray diffraction (XRD).^46^ This method probes the crystallinity of intermediates and products in real time which enables better tuning of reaction conditions. In a previous work, we demonstrated that a mechanochemically synthesized COF can effectively adsorb PFAS from water.^43^ Here we report the synthesis of three COFs designed to investigate how pore wall modification within the framework dictates PFAS adsorption behavior (Fig. 1). The three isoreticular COFs were synthesized mechanochemically. They differ only in their pore wall functionality. Under the investigated conditions, the fluorinated COF exhibited higher adsorption than the methylated and unsubstituted analogues. This difference is attributed to the greater electronegativity and hydrophobicity of its pore walls.^47,48^

**Fig. 1:**
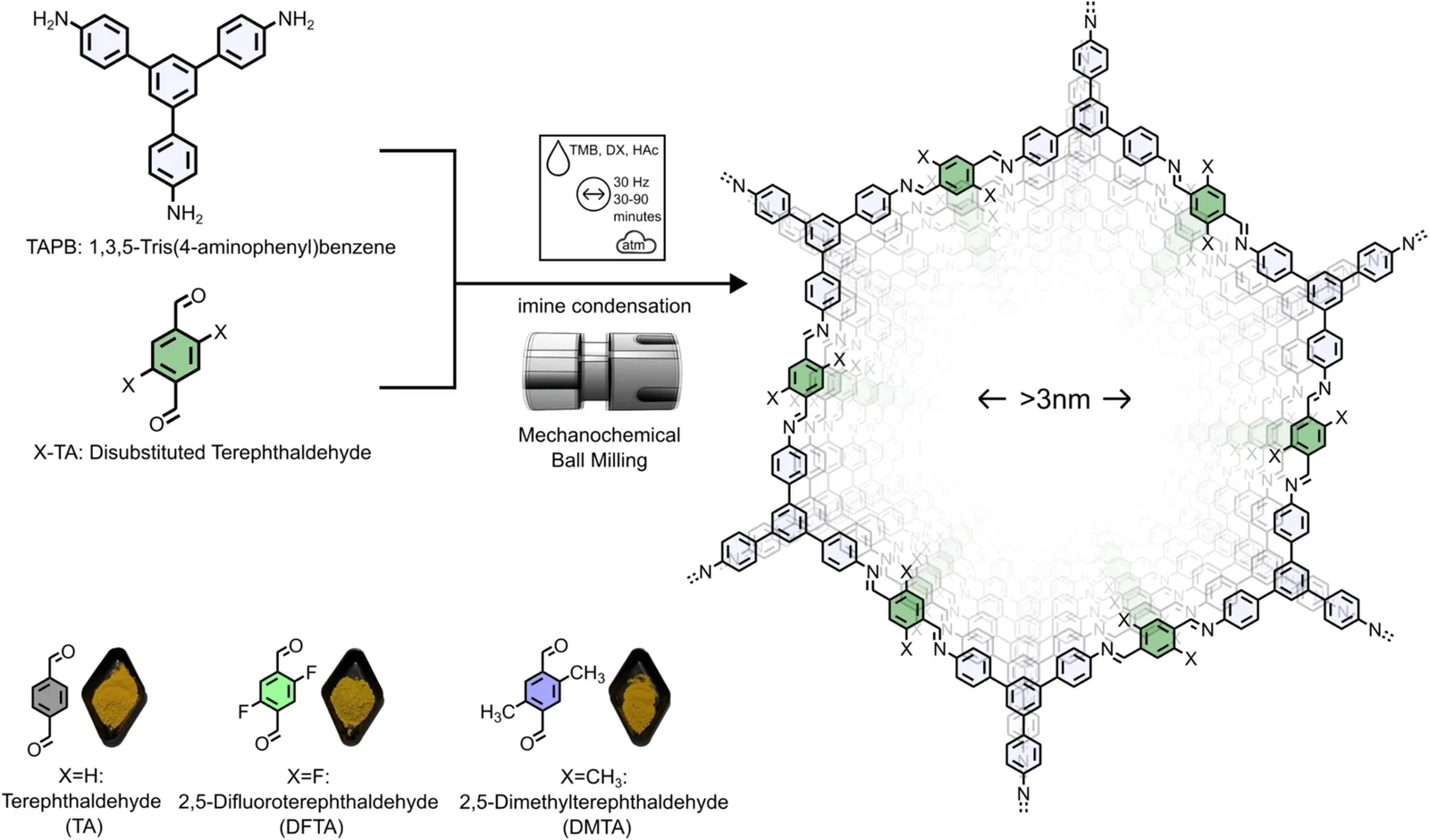
Schematic representation of the mechanochemical synthesis of TAPB-X-TA COF from 1,3,5-tris(4-aminophenyl)benzene (TAPB) and disubstituted terephthaldhyde (X-TA), displaying the COF's hexagonal pore structure and design of an isoreticular series by modifying X to H (terephthaldehyde), F (2,5-difluoroterephthaldehyde) and CH_3_ (2,5-dimethylterephthaldehyde).

## Results and discussion

Three COFs were synthesized *via* mechanochemical imine condensation of an 1,3,5-tris(4-aminophenyl)benzene (TAPB) and the corresponding terephthaldehyde (X-TA) linker. The reaction of TAPB with unsubstituted terephthalaldehyde (TA), 2,5-difluoroterephthalaldehyde (DFTA), and 2,5-dimethylterephthalaldehyde (DMTA) afforded TAPB-TA, TAPB-DFTA, and TAPB-DMTA COFs, respectively. The reaction parameters were optimized for a horizontal ball mill. Equimolar amounts of reagents were placed in a 10 mL stainless steel jar with mesitylene, 1,4-dioxane and glacial acetic acid as liquid additives. OptimaI results were achieved by milling with two 5 mm balls at 30 Hz frequency. Reaction times and volume of liquid were optimized individually for each COF. Further information can be found in the SI. These COFs are already reported in literature,^49–62^ however, to our knowledge, this is the first report of any of these COFs being synthesized using mechanochemistry.

The successful synthesis of the COFs *via* imine condensation was confirmed by Fourier-transform infrared (FT-IR) spectroscopy (Fig. 2a and S1, SI). The appearance of a new imine (C=N) stretching vibration at 1618 cm^−1^ for TAPB-TA and TAPB-DMTA COFs, and at 1620 cm^−1^ for TAPB-DFTA COF, indicates the formation of the imine linkage.^49–62^ The slight upshift observed for the fluorinated COF reflects the electronic influence of fluorine on the COF's vibrational properties.^63^ The C=O stretching band of residual aldehydes at 1695 cm^−1^ can still be detected as a weak band in the methyl- and fluorine-substituted COFs. In contrast, the unsubstituted TAPB-TA COF shows a more prominent aldehyde signal, indicating residual aldehyde groups. This suggests that defects are formed during the mechanochemical synthesis. The full range of FT-IR results are shown in Fig. S1 of SI.

**Fig. 2:**
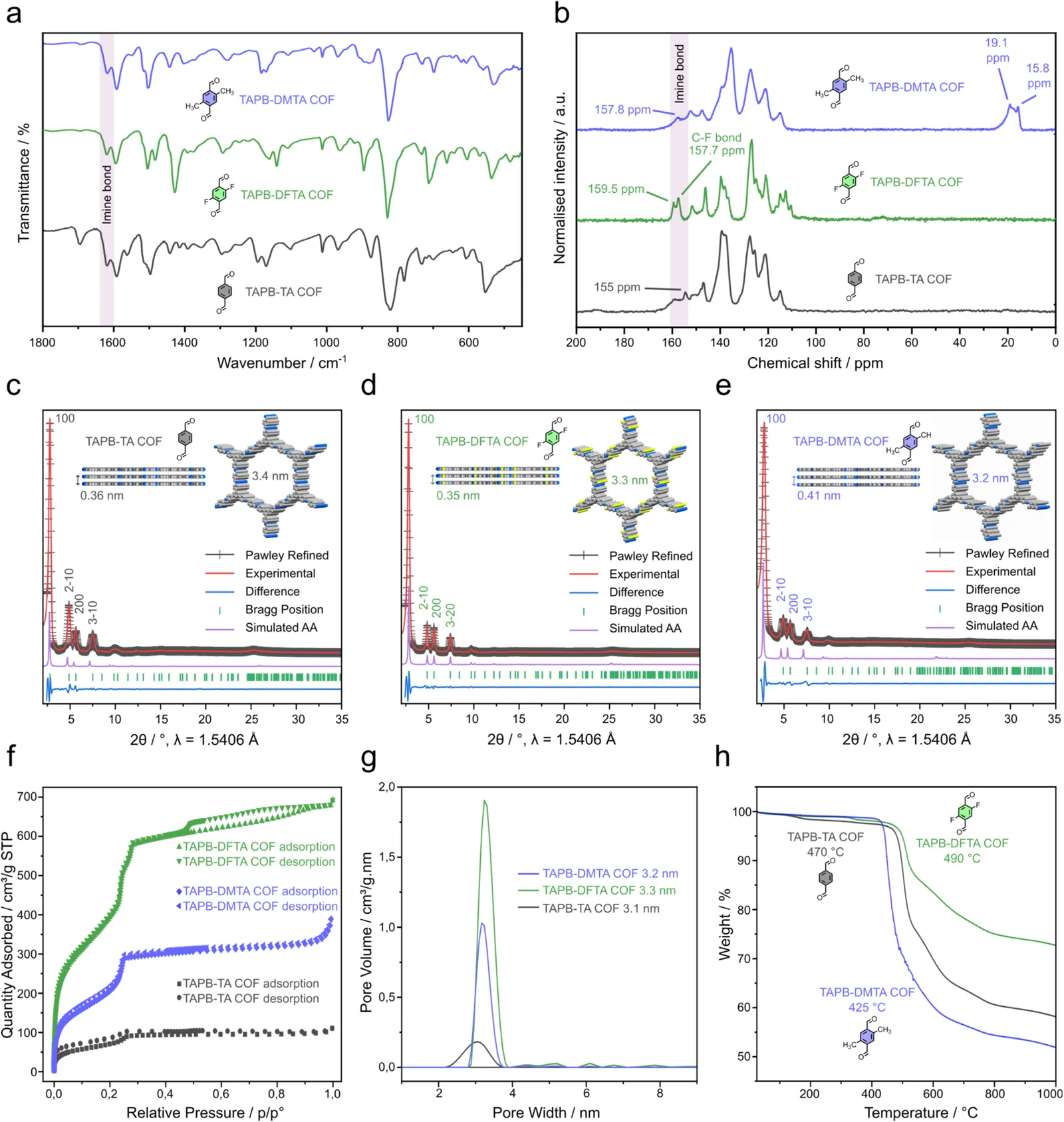
(a) FT-IR spectra of TAPB-TA COF, TAPB-DFTA COF and TAPB-DMTA COF highlighting the common imine C=N stretch and spectral differences from distinct functional groups. (b) ^13^C ssMAS NMR spectra of the three COFs (c) Pawley refinement of XRD pattern of TAPB-TA COF showing simulated crystal structure with pore and interlayer spacing (d) Pawley refinement of TAPB-DFTA COF XRD pattern. (e) Pawley refinement of TAPB-DMTA COF XRD pattern. (f) N_2_ adsorption–desorption isotherms revealing TAPB-DFTA COF exhibits the highest N_2_ uptake capacity (g) pore size distributions derived from N_2_ adsorption data and (h) thermogravimetric analysis (TGA) curves demonstrating TAPB-DFTA COF displays superior thermal stability.

Solid-state ^13^C magic angle spinning nuclear magnetic resonance (ssMAS NMR) provides further confirmation of a successful COF formation through the characteristic imine (C=N) carbon resonances (Fig. 2b). Despite this shared linkage, the imine peak positions and multiplicities reveal distinct chemical environments driven by linker substitution. TAPB-TA COF shows an imine peak at 155 ppm.^64^ TAPB-DFTA COF exhibits a signal for C=N at 159.5 ppm and a signal for C–F at 157.7.^63^ TAPB-DMTA COF displays an imine peak at 157.8 ppm.^65^ It also shows a split peak at 19.1 and 15.8 ppm corresponding to the methyl functional group. Kang *et al*.^66^ have demonstrated that the peaks for methyl substituents can serve as sensitive probes that reveal the COF's stacking architecture. The upfield-shifted signal at 15.8 ppm can be attributed to methyl groups experiencing interlayer interactions in AA stacking structures, where adjacent COF layers are aligned directly on top of each other. In this closely packed geometry, methyl carbons are shielded by the enhanced electron density from neighbouring layers, resulting in the characteristic upfield shift. Conversely, the peak at 19.1 ppm corresponds to methyl groups in non-AA stacking structures including offset stacking and random arrangements. These methyl carbons are more deshielded and thus appear downfield. XPS spectra further validate the successful formation of the imine-linked COFs (Fig. S2, SI). All COFs exhibit a characteristic N 1s peak at 398.5 eV, corresponding to imine nitrogen, along with a small hump at higher binding energy attributed to surface amine species and slight surface oxidation. In the case of DMTA, the C 1s peak at 284 eV shows higher intensity compared to the others due to the presence of methyl groups. DFTA displays a characteristic F 1s peak at 689 eV.

Powder XRD analysis confirmed the crystallinity of the COFs (Fig. 2d–f and S3, SI). The diffraction patterns exhibited a prominent reflection at 2*θ* = 2.8° (*λ* = 1.5406 Å), corresponding to the (100) plane. Although this first reflection appears near 2.8° in all cases, subtle differences were observed among the three structures. Specifically, TAPB-TA COF showed the first reflection at 2.78°, TAPB-DFTA COF at 2.80°, and TAPB-DMTA COF at 2.83°. These slight shifts may be attributed to the increasing size of the functional groups.^67,68^ Additional reflections were observed at 4.9° and 5.6° corresponding to the (21̄0) and (200) planes, respectively.^69^ Using these experimental data, 2D structures were proposed with inclined AA stacking using Materials Studio (Fig. 2d–f and S4–S6, SI). All structures crystallise in the hexagonal space group *P*6̄ (no. 174) Pawley refinement confirmed excellent agreement between experimental and simulated patterns with *R*_p_ values of 4.9%, 6.1% and 5.4% for TAPB-TA COF, TAPB-DFTA COF and TAPB-DMTA COF, respectively. The π–π stacking distance between the layers was calculated to be 0.36 nm for TAPB-TA COF, 0.35 nm for TAPB-DFTA COF and 0.41 nm for TAPB-DMTA COF.

Nitrogen adsorption measurements revealed typical type IV isotherms for all COFs (Fig. 2f and S7–S9, SI). The BET surface areas were 262.0 ± 2.7 m^2^ g^−1^ for TAPB-TA COF, 1237.0 ± 3.7 m^2^ g^−1^ for TAPB-DFTA COF, and 592.5 ± 6.6 m^2^ g^−1^ for TAPB-DMTA COF, with corresponding DFT pore sizes of 3.1, 3.3, and 3.2 nm (Fig. 2g), respectively. Despite having isoreticular structures, the three COFs have markedly different BET surface areas. This highlights the significant impact of linker substituents on mechanochemical assembly, pore stability and activation behaviour. In TAPB-DFTA, the small, electron-withdrawing fluorine atoms promote more ordered stacking and suppress defect-induced pore collapse, resulting in the highest accessible porosity. In TAPB-DMTA, the methyl groups introduce steric hindrance, which only partially stabilises the pore network and restricts accessible porosity. In contrast, the unsubstituted TAPB-TA shows the lowest surface area and the smallest pore size, even smaller than the one in the simulated structure. We attribute this to a higher defect density, as indicated by FT-IR. Consequently, parts of the framework remain inaccessible to nitrogen, leading to a reduced BET surface area.^70–73^

Thermogravimetric analysis (TGA) of the COFs revealed high thermal stability, though notable differences were observed among them (Fig. 2i). Among the three, TAPB-DFTA COF demonstrated the highest thermal robustness. Decomposition initiates around 490 °C and it retains 73% of its mass at 1000 °C. In contrast, TAPB-DMTA COF exhibited the lowest thermal stability. It begins to decompose near 425 °C and retains only 52% of its mass at 1000 °C. TAPB-TA COF displayed intermediate behaviour. This trend in thermal stability TAPB-DFTA > TAPB-TA > TAPB-DMTA can be attributed again to the nature of the substituents on the terephthaldehyde linker. It has been reported that compact, polar substituents such as fluorine generally enhance COF stability, while larger, nonpolar alkyl groups tend to compromise it.^74–77^ The unsubstituted TA linker in TAPB-TA COF, lacking both the stabilizing effects of fluorine and the destabilizing influence of methyl groups, contributes to its moderate thermal performance (Fig. 2c–e).

To investigate COF formation in real time, TRIS XRD was performed at the HZB BESSY II synchrotron facility. Transparent PMMA jars were used to ensure unobstructed X-ray transmission with minimal attenuation. Similar to the optimized synthesis, equimolar amounts of reagents were mixed with mesitylene, dioxane and acetic acid. However, no differences were observed between reactions, as COF formation began almost immediately (Fig. S10, SI). This interfered with our ability to monitor the formation process. We learnt from *ex situ* experiments that acetic acid is the key additive driving the reaction. The optimized synthesis uses glacial acetic acid (17.4 M) which accelerates COF formation significantly. Its drawback, however, is that it renders the reaction too fast to follow at the synchrotron. For this reason, 6 M acetic acid was subsequently used to slow the process.

In the newly optimized slower synthesis (Fig. 3), the first framework reflections for TAPB-TA COF appeared after 6 minutes of milling which was marked by the emergence of a peak at *q* = 0.2 (2*θ* ≈ 2.8°). Framework formation in TAPB-DFTA and TAPB-DMTA COFs was faster, initiating within the first and second minute, respectively. Interestingly, TAPB-TA COF exhibited an additional reflection at *q* = 0.27 (2*θ* ≈ 3.5°). This peak emerged after one minute of milling and vanished after 18 minutes. No such reflection appeared in the other two COFs or in any *ex situ* XRD data, including reactions quenched after 5 seconds or material ground manually for 5 seconds. This suggests the peak possibly corresponds to an intermediate or a cocrystal that is short-lived or highly sensitive to environment. On the other hand, reflections from the reactants did not fully disappear *in situ* which suggests incomplete consumption. However, *ex situ* XRD patterns showed no residual reactant reflections, implying that the remaining *in situ* signals likely originate from material sticking to the jar walls (Fig. 4).

**Fig. 3:**
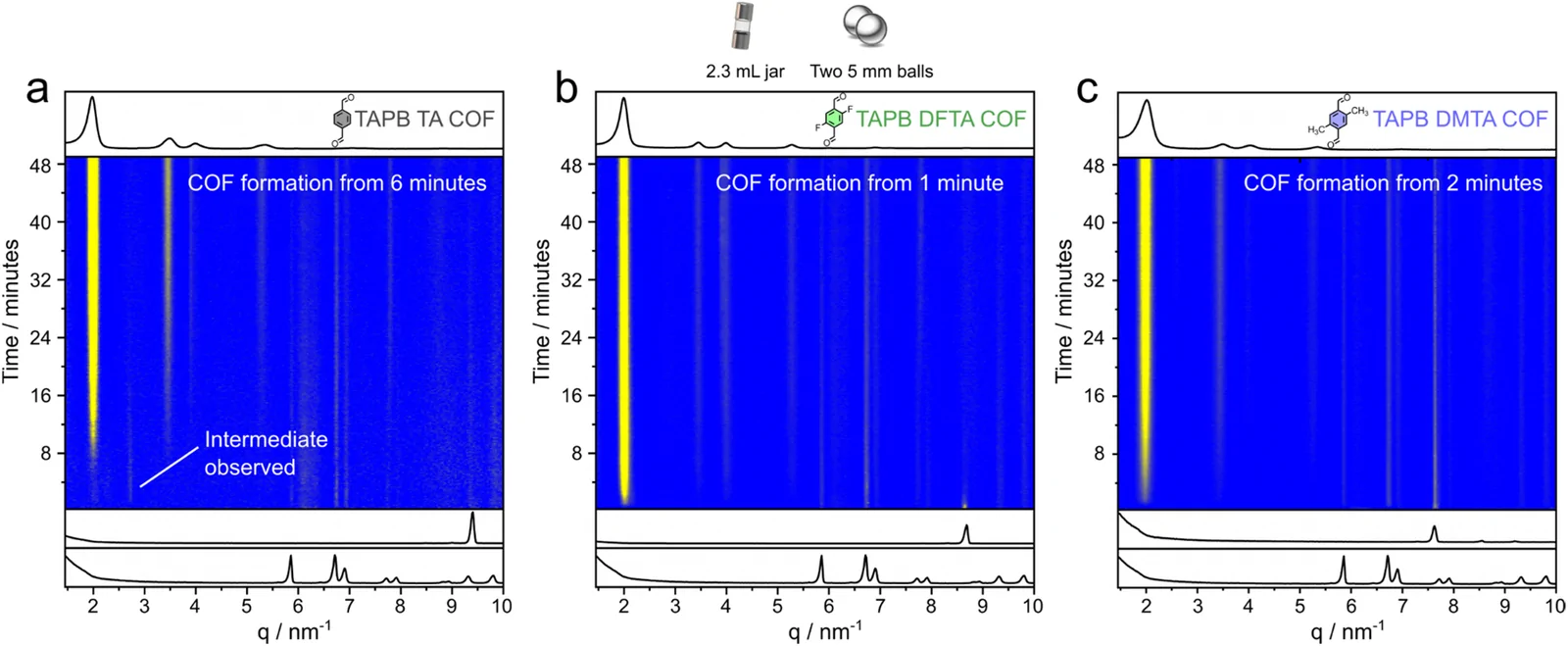
Time-resolved *in situ* synchrotron X-ray diffraction profiles for (a) TAPB-TA COF, (b) TAPB-DFTA COF, and (c) TAPB-DMTA COF, demonstrating rapid framework formation within the first 1–6 minutes of reaction, followed by intensification of COF reflections. Weak reagent reflections persist throughout the reaction but remain low in intensity.

**Fig. 4:**
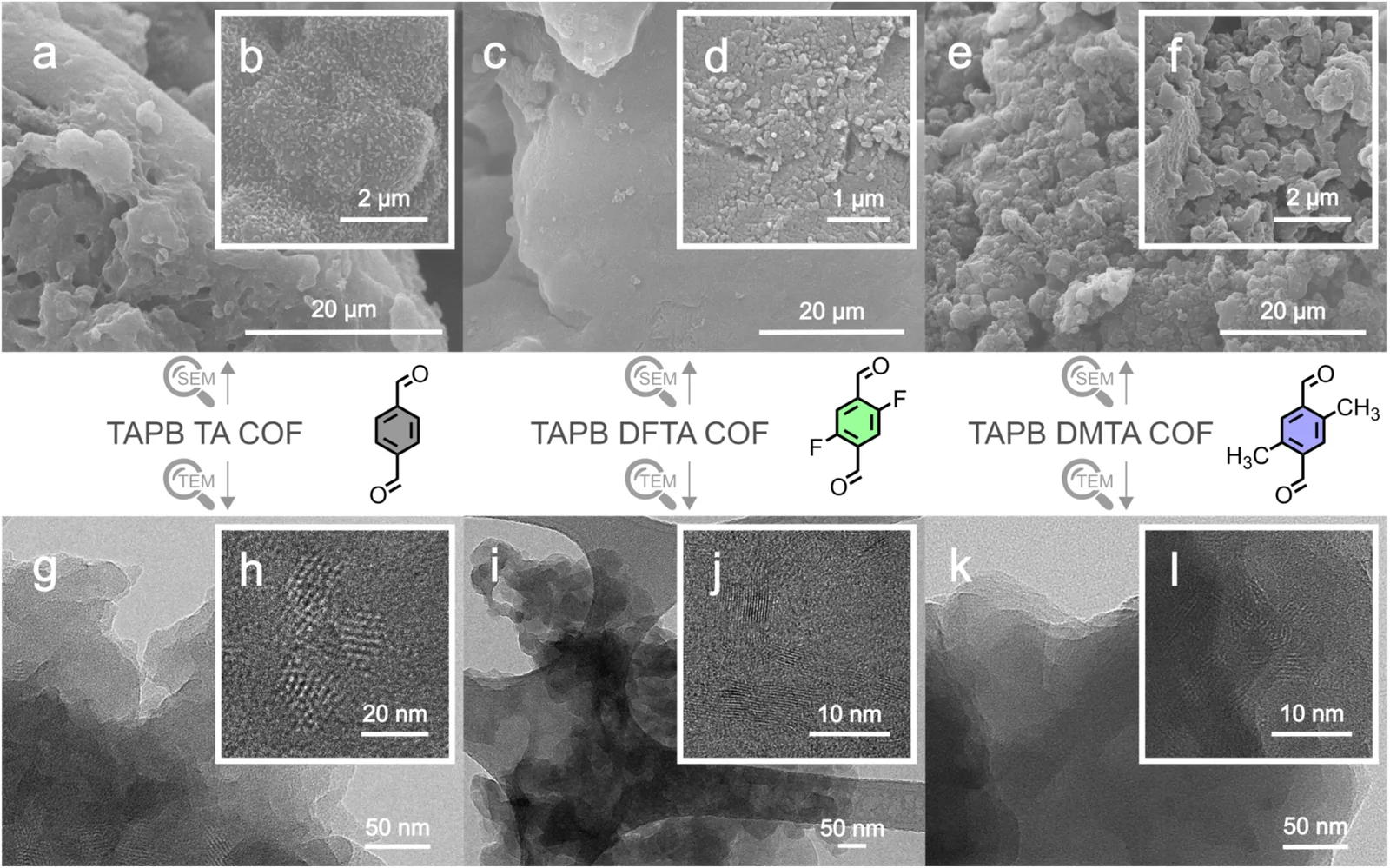
(a–f) Scanning electron microscopy (SEM) images of the three COFs, TAPB-TA, TAPB-DFTA, and TAPB-DMTA, showing their respective morphologies and surface features. (g–l) Transmission electron microscopy (TEM) images of the same series of COFs revealing their structure like crumpled paper and showing crystallinity at higher magnification.

SEM analysis of the three COFs revealed aggregates composed of numerous small, irregularly shaped particles, likely formed due to the high mechanical stress during synthesis. Based on TEM images, the three COFs look similar. They appear as disordered layers, resembling crumpled paper. Under high magnification, crystallinity is visible in the form of parallel, regular lines.

The three COFs were evaluated for their capacity to adsorb perfluoroalkyl substances (PFAS), specifically perfluorooctanoic acid (PFOA) and perfluorooctane sulfonic acid (PFOS). Before testing, stability assessments were performed to confirm structural integrity of the COF (Fig. 2d–f and S11, SI). XRD and FT-IR confirms the structural stability of the COFs in different environments. Following the stability tests, adsorption experiments were performed. In each experiment, 10 mg of COF was suspended in different concentrations of 25 mL of PFAS solution and agitated on an orbital shaker. After adsorption, the suspension was filtered and the residual PFAS concentration in the filtrate was determined using ultra-high-performance liquid chromatography (UHPLC) coupled with mass spectrometry. Adsorption efficiency was then calculated based on the ratio of the initial to the final PFAS concentrations.

Adsorption kinetics were investigated first. The experiments were conducted at contact times of 10, 30, and 60 minutes, as well as 2 hours. These time stamps were chosen based on previous studies^43^ showing that PFAS adsorption on porous materials occurs rapidly, typically within minutes. The initial PFAS concentration was approximately 0.1 mg L^−1^ (100 µg L^−1^) for both PFOA and PFOS. The results showed that the three COFs adsorbed a significant portion of both PFAS within the first 10 minutes, approaching equilibrium shortly afterwards (Fig. 5). The three COFs showed similar performance for PFOS, with more than 98% adsorption within 10 minutes and negligible differences among them. In contrast, differences were observed for PFOA. TAPB-TA COF showed 87.1 ± 3.7% adsorption while TAPB-DFTA COF showed 94.6 ± 0.4%. TAPB-DMTA COF demonstrated 78.8 ± 1.6% adsorption after 2 hours of adsorption. The difference in adsorption of PFOA and PFOS is consistent with literature that observed a lower interaction of PFOA with frameworks compared to PFOS.^78^ Among the COFs, TAPB-DFTA COF exhibited the highest PFOA adsorption, while TAPB-DMTA performed the worst. Kinetic analysis suggested the adsorption followed a pseudo second order model, indicating that chemisorption governs the rate limiting step (Fig. S12, SI).

**Fig. 5:**
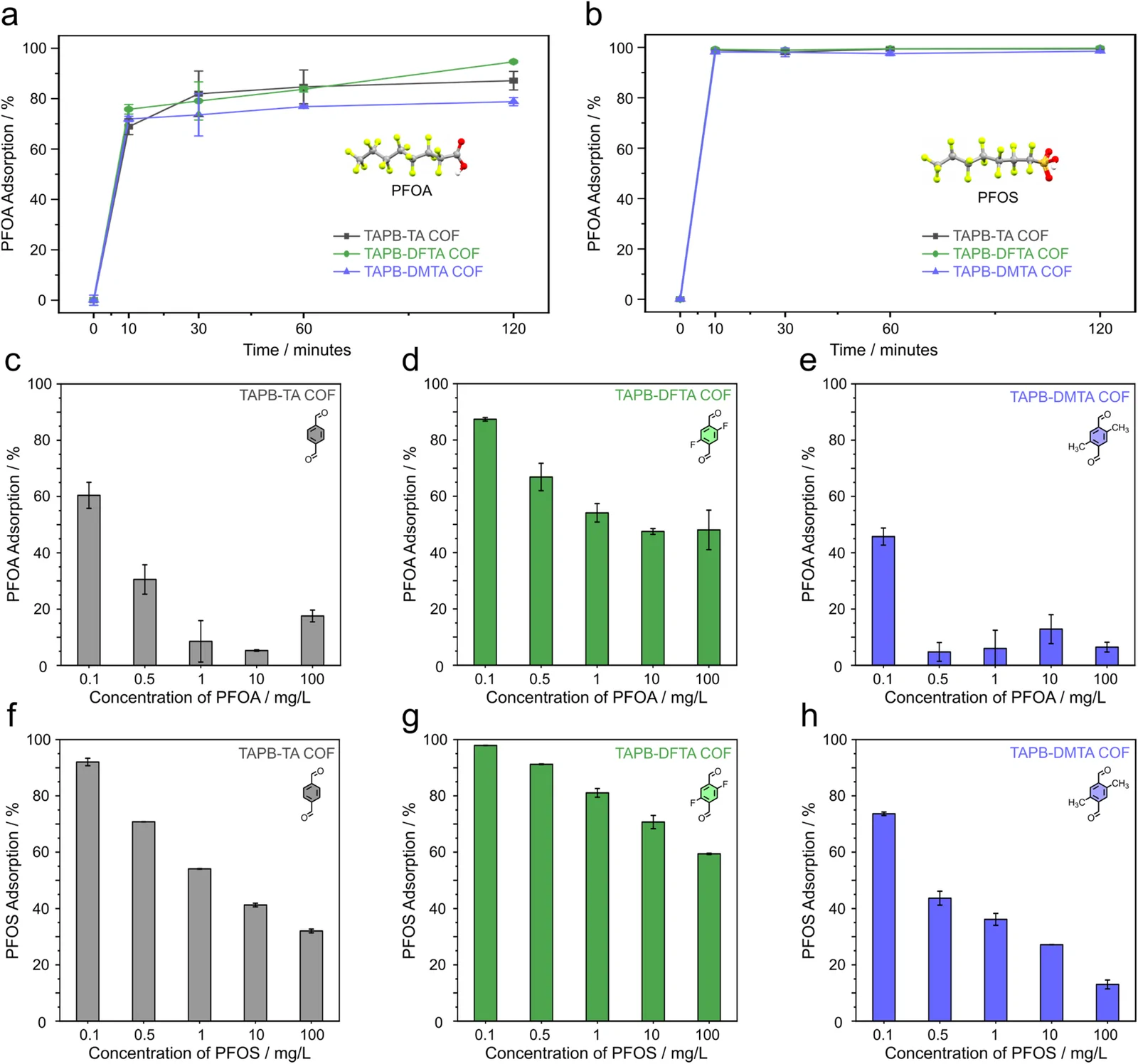
(a) Kinetic PFOA adsorption data of TAPB-TA COF, TAPB-DFTA COF and TAPB-DMTA COF. TAPB-DFTA COF was the best performer (b) kinetic PFOS adsorption data of TAPB-TA COF, TAPB-DFTA COF and TAPB-DMTA COF. TAPB-DFTA COF was the best performer here as well (c) equilibrium PFOA adsorption data of TAPB-TA COF at different concentrations of PFOA (d) equilibrium PFOA adsorption data of TAPB-DFTA COF at different concentrations of PFOA (e) equilibrium PFOA adsorption data of TAPB-DMTA COF at different concentrations of PFOA (f) equilibrium PFOS adsorption data of TAPB-TA COF at different concentrations of PFOS (g) equilibrium PFOS adsorption data of TAPB-DFTA COF at different concentrations of PFOS (h) equilibrium PFOS adsorption data of TAPB-DMTA COF at different concentrations of PFOS.

Additional equilibrium adsorption experiments were performed at elevated PFAS concentrations, ranging from 0.1 mg L^−1^ to 100 mg L^−1^. Across the full concentration range, the same overall trend was observed. TAPB-DFTA COF exhibited the highest adsorption capacity, followed by TAPB-TA COF, with TAPB-DMTA COF showing the lowest performance in comparison. Notably, at the highest concentration tested (100 mg L^−1^), substantial differences in adsorption efficiencies were seen. TAPB-DFTA COF adsorbed 48.1 ± 7.0% of PFOA, whereas TAPB-TA COF and TAPB-DMTA COF achieved only 17.6 ± 2.1% and 6.5 ± 1.7%, respectively. A similar pattern was observed for PFOS at 100 mg L^−1^, with TAPB-DFTA COF removing 59.4 ± 0.2%, while TAPB-TA COF and TAPB-DMTA COF adsorbed 32.0 ± 0.7% and 13.0 ± 1.6%, respectively. Similar trend was also observed for GEN X and PFBS (Fig. S13, SI)

TAPB-DFTA COF performed the best probably because fluorinated pores create a highly favorable microenvironment for PFAS. The perfluoroalkyl tail exhibits strong fluorophilic affinity for the difluorinated linker through F⋯F interactions, creating a low polarity, water-excluding pore interior that drives PFOA from solution.^79^ Fluorine atoms minimize steric hindrance, maximize pore accessibility and reduce water competition for adsorption sites.^80^ Although the unsubstituted TAPB-TA COF exhibited a lower BET surface area than the methyl-functionalized TAPB-DMTA COF, it unexpectedly displayed a higher adsorption capacity. To investigate this unexpected behavior, small angle X-ray scattering (SAXS) measurements were performed (Fig. S14). The SAXS profile of TAPB-TA, in comparison, exhibits broader and less intense diffraction peaks at low *q*, originating from the periodic pore structure, together with form factor scattering at higher *q* and the absence of distinct diffraction peaks in this region. These features indicate a lower degree of long-range structural order compared to the functionalized COFs. While the measured *q* range does not allow a quantitative determination of the external surface area, the reduced structural order is consistent with the formation of smaller particles, which may provide a greater accessible external surface for adsorption. In contrast, as previously mentioned, ssMAS NMR revealed the coexistence of AA and AB layer stacking in TAPB-DMTA.^66^ The presence of mixed stacking likely interrupts the pore channels, reduces pore accessibility and hinders guest transport through the framework. Together, these observations provide a plausible explanation for why the unsubstituted TAPB-TA COF outperformed the methyl-functionalized analogue despite its lower BET surface area.

To understand the adsorption between the PFAS and the COF, FT-IR spectra of the COFs were compared before and after adsorption. As shown in Fig. S15 in SI, the characteristic imine stretching bands remain present after PFOS adsorption, indicating that the imine linkages within the COF frameworks remain structurally intact. However, a slight broadening of the imine band is observed following adsorption, suggesting an interaction between the imine functionalities and the adsorbed PFOS molecules. In our previous work, such spectral broadening has previously been associated with hydrogen bonding and electrostatic interactions involving protonated imine sites.^43^ Additionally, the appearance of vibrational bands corresponding to CF_2_ groups after adsorption provides spectroscopic evidence for the presence of PFAS within the COF framework.

## Conclusion

The mechanochemical synthesis of the TAPB-X-TA COF series provides a green and robust method for producing large-pore imine frameworks with pore-wall modifications for the adsorption of PFAS from water. Linker fluorination and methylation markedly influence interlayer stacking, accessible porosity and thermal robustness across the three materials, with TAPB-DFTA COF exhibiting the highest crystallinity, stability and BET surface area. TRIS XRD reveals that the three COFs form rapidly under mechanochemical conditions. Batch adsorption experiments conducted over a broad range of PFAS concentrations demonstrate fast kinetics and near-quantitative PFOS removal for the three COFs. TAPB-DFTA COF, however, consistently exhibits superior uptake of PFOA, PFOS and shorter-chain analogues. The combination of a high surface area and fluorinated pore walls in TAPB-DFTA creates a hydrophobic and fluorophilic environment that enhances PFAS binding cooperatively. PFAS uptake proceeds through a synergistic interplay of hydrophobic, electrostatic and hydrogen-bonding interactions between anionic head groups and protonated imine functionalities.

Taken together, these results demonstrate that mechanochemical synthesis combined with targeted pore-wall modification is a powerful and sustainable strategy for tailoring the structure and pore chemistry of COFs for PFAS adsorption. In particular, the fluorinated TAPB DFTA COF showed higher PFAS removal than the non-fluorinated and methyl functionalized COFs.

## Conflicts of interest

There are no conflicts of interest to declare.

## Supplementary Material

RA-016-D6RA06859F-s001

## Data Availability

Data for this article are available at Zenodo at https://doi.org/10.5281/zenodo.19091948. Supplementary information (SI): experimental procedures, additional characterization data for the synthesized COFs and supporting data for the PFAS adsorption and stability studies. See DOI: https://doi.org/10.1039/d6ra06859f.
