# Supplementary material for: Fluorinated pore walls boost PFAS capture in mechanochemically synthesized covalent organic frameworks
Source: RSC Adv. 2026 Oct 9;16(47):61821–31. doi: 10.1039/d6ra06859f (PMC13650538; doi:10.1039/d6ra06859f)
Supplement: Supplementary file 1 [file RA-016-D6RA06859F-s001.pdf]

Supplementary Information (SI) for RSC Advances.
This journal is © The Royal Society of Chemistry

# Supporting Information

# Fluorinated Pore Walls Boost PFAS Capture in Mechanochemically Synthesized Covalent Organic Frameworks

Maroof Arshadul Hoque[a,b], Tanja Westphalen[a], Stephanie Kluge[a], Christian Piechotta[a], Jana Falkenhagen[a], Nader de Sousa Amadeu[a], Carsten Prinz[a], Prasenjit Das[c], Ernesto Scoppola[d], Tomasz M Stawski[a], Biswajit Bhattacharya[a*], Franziska Emmerling[a,b*]

[a] BAM Federal Institute for Materials Research and Testing
Richard-Willstätter-Str. 11, 12489 Berlin (Germany)
Email- franziska.emmerling@bam.de

[b] Department of Chemistry,
Humboldt University Berlin
Brook-Taylor-Straße 2, 12489 Berlin (Germany)

[c] Department of Chemistry, Functional Materials,
Indian Institute of Technology Ropar,
SSB Block, Rupnagar, Punjab, 140001 (India)

[d] Department of Biomaterials,
Max Planck Institute of Colloids and Interfaces,
Am Mühlenberg 1, 14476 Potsdam (Germany)

## Abstract

Per- and polyfluoroalkyl substances (PFAS) are persistent water contaminants that require robust, high-affinity sorbents. Covalent organic frameworks (COFs) provide chemically stable, modular platforms for capturing PFAS and mechanochemical synthesis offers an environmentally friendly, scalable method of production. In this study, we use mechanochemistry to quickly produce an isoreticular series of large-pore imine COFs (TAPB-TA, TAPB-DFTA and TAPB-DMTA) with systematically varied pore-wall functionality, which is optimised for PFAS adsorption. The fluorinated TAPB-DFTA COF exhibits the greatest crystallinity, the highest BET surface area (1237 $m^2$ $g^{⁻1}$) and the greatest thermal stability. These properties result in superior uptake of PFOA, PFOS and short-chain analogues compared to the methylated and unsubstituted analogues in batch adsorption experiments across a broad concentration range. The three COFs achieve near-quantitative PFOS removal within minutes,

while TAPB-DFTA maintains significantly higher PFOA removal even at a feed concentration of 100 mg/L. These findings indicate that mechanochemical synthesis combined with pore wall modification offers a possible approach for tuning COF adsorption sites for PFAS uptake.

## Table of Contents


| | |
|---|---|
| S1. Chemicals | 3 |
| S2. Synthesis | 3 |
| S3. Characterization methods | 4-7 |
| S4. Characterization of COFs | 8-32 |
| S4.1 FTIR analysis | 8-9 |
| S4.2 XPS analysis | 10 |
| S4.3 XRD analysis | 11 |
| S4.4 Simulated structures | 12-13 |
| S4.5 Nitrogen adsorption | 14-17 |
| S4.6 TRIS XRD analysis | 18 |
| S4.7 Stability tests | 19 |
| S4.8 PFAS adsorption data | 20-30 |
| S4.9 Correlation of SAXS analysis with PFAS adsorption | 31-32 |
| S4.10 FT-IR analysis of COF before and after adsorption | 32 |

## S1. Chemicals

1,3,5-tris(4-aminophenyl)benzene (TAPB) with purity 97%, 2,5-dimethylterephthaldehyde (DMTA) with purity 95% and 2,5-difluoroterephthaldehyde (DFTA) with purity 97% were purchased from BLD Pharmatech GmbH. Terephthaldehyde (TA) with purity 99% was bought from Sigma Aldrich. The chemicals mesitylene (98% purity), acetic acid (99.8%) and methanol (99.8%) were ordered from TH Geyer GmbH und Co. KG. Methanol (≥99.9%) of PFAS grade for LC-MS was obtained from the same source for use in UHPLC MS measurements. All chemicals were used in their original state, without undergoing any further purification processes.

## S2. Synthesis

TAPB-TA COF was synthesized via a mechanochemical approach. A 10 mL stainless steel milling jar was filled with TAPB (105 mg), TA (40 mg), and two 5 mm stainless steel balls. Mesitylene, 1,4-dioxane and glacial acetic acid (150 μL each) were added using a pipette to facilitate liquid-assisted grinding. The mixture was milled in a RETSCH MM 400 vibratory ball mill at a frequency of 30 Hz for 30 minutes.

TAPB-DFTA COF was prepared using a similar protocol, with TAPB (105 mg) and DFTA (51 mg) in a 10 mL jar containing two 5 mm balls. The same volume of liquid additives (150 μL each) was used but the milling time was extended to 60 minutes to ensure complete imine condensation and framework formation.

TAPB-DMTA COF was synthesized under comparable conditions, using TAPB (105 mg) and DMTA (49 mg) with two 5 mm stainless steel balls in a 10 mL jar. The milling time was maintained at 30 minutes but the volume of liquid additives was reduced to 70 μL each to account for the solubility and reactivity of the DMTA linker.

After milling, the COF powders were dried under a fume hood for 1 h and purified by Soxhlet extraction with methanol for 24 h to remove residual monomers. The extracted samples were then oven dried at 60 °C for 6 h. Before the adsorption experiments, the COFs were activated under vacuum overnight at 60 °C.

## S3. Characterization methods

### S3.1 Fourier Transform Infrared Spectroscopy (FT-IR)

FT-IR spectra were acquired using a Nicolet 670 FT-IR spectrometer (ThermoFisherScientific GmbH) in attenuated total reflection (ATR) mode with a "Golden Gate" sample holder. The holder was cleaned with ethanol and acetone after every measurement, and the background spectrum was taken in air. Each spectrum involved 32 scans at 4 $cm^{-1}$ resolution, captured in transmission mode over 4000-400 $cm^{-1}$, with instrument control via OMNIC software. All spectra underwent identical mathematical processing and were plotted using Origin software.

### S3.2 Solid state magic angle spinning nuclear magnetic resonance spectroscopy (ssMAS NMR)

ssMAS NMR spectra were recorded at a Bruker AVANCE Neo spectrometer under the field of 9.4 T (400 MHz for 1H) at room temperature. 1.9 mm MAS zirconia rotors were spun along the magic angle at speeds between 12 and 36 kHz (higher rotations for more resolution, lower rotations for more effective cross-polarization). Cross-polarization from 1H to 13C was achieved with contact times between 3 and 5 ms, 1H decoupling was done with spinal64 and under 100 kHz fields during typical acquisition times or 15 ms. Repetition times were 5 s.

### S3.3 X-ray diffraction (XRD)

Powder X-ray diffraction was performed using a D8 Advance Diffractometer (Bruker AXS, Karlsruhe, Germany) equiped with an energy-dispersive LynxEye XE-T detector. Measurements used a copper X-ray source (wavelength 1.54178 Å) without a monochromator, in Bragg-Brentano geometry at 40 kV and 40 mA. Parameters included a step size of 0.02° and 1 s per step. Data evaluation was done with DIFFRAC.EVA software and plots were generated using Origin.

## S3.4 Thermogravimetric analysis (TGA)

TGA analysis was performed on a METTLER Toledo TGA/DSC 3+ instrument. COF samples were heated from 25 °C to 950 °C in an open 70 µL aluminium oxide crucible under a nitrogen flow of 80 mL/min. The heating rate was 10 °C/min and finally, the data obtained was evaluated and plotted with Origin software.

## S3.5 X-ray Photoelectron Spectroscopy (XPS)

X-ray Photoelectron Spectroscopy (XPS) was performed using a K-Alpha™ + system (Thermo Scientific) equipped with a hemispherical 180° dual-focus analyzer and 128-channel detector. Micro-focused Al-Kα radiation from the X-ray monochromator was employed. Powder COF samples were pressed onto carbon tape and mounted on the sample holder. Data was acquired with a 400 µm X-ray spot size, 20 scans for the survey and 50 scans for regions. The data obtained was evaluated and plotted using Origin software.

## S3.6 Nitrogen adsorption

Nitrogen adsorption/desorption isotherms were measured on a Micromeritics TriFlex surface area analyzer at 77 K, maintained by a liquid nitrogen bath. Samples of approximately 200 mg COF were degassed for 12 hours at 120 °C under dry nitrogen flow prior to analysis. Specific surface area ($m^2$ $g^{-1}$) was calculated via the Brunauer-Emmett-Teller (BET) model and pore width was determined from the DFT pore volume plot. Data evaluation used MicroActive software from Micromeritics Instrument Corporation followed by plots generated in Origin.

## S3.7 Time resolved *in situ* X-ray diffraction (TRIS XRD)

*In situ* ball milling experiments were carried out using a Fritsch Pulverisette 23 shaker mill featuring vertical motion. The mill provides a fixed 9 mm amplitude and variable frequency from 15 to 50 Hz, along with an adjustable timer. It employed a 2.3 mL jar made of three parts: two stainless steel end pieces and a transparent Perspex middle section 0.75 mm thick, with overall dimensions of 40 mm length and 12 mm internal diameter. TRIS XRD data was acquired at µSpot (BESSY-II, Helmholtz Zentrum Berlin) using a low-energy 17 keV incident beam of 150 µm diameter and an Eiger 9 M 2D detector. Data collection occurred every 10 seconds during mill operation with a sample-to-detector distance of approximately 250 mm. Two-dimensional diffraction images were integrated into conventional one-dimensional XRD patterns via an in-house Python script.

Background subtraction was performed using another in-house Python script followed by plotting the data in contour plots in Origin.

### S3.8 Scanning electron microscopy (SEM)

Samples were examined using scanning electron microscopy (SEM) on an FEI XL30 ESEM equipped with a tungsten cathode, SE/GSED/BSE detectors, high- and low-vacuum modes, an energy-dispersive X-ray spectrometer SDD Quantax 200, and a cryotransfer system Alto 1000. Prior to imaging, samples received a thin gold coating to enable electrical conduction.

### S3.9 Transmission electron microscopy (TEM)

Transmission electron microscopy (TEM) images were obtained with a Talos F200S Microscope (Thermo Fisher Scientific) at an accelerating voltage of 200 kV. The method transmits a high-energy electron beam through the specimen to produce high-resolution images. Images were captured in TEM mode using a Ceta 16M camera. For preparation, 10 $\mu$L of sample solution (1 mg sample in 0.2 mL water) was deposited on a 3 mm copper grid (lacey, 400 mesh) and air-dried at room temperature. Imaging and data analysis utilized Velox software (version 3.3).

### S3.10 PFAS adsorption tests

PFAS solutions with a concentration of 100 mg/L were prepared either by dissolving PFAS powder (PFOA and PFOS) in MilliQ water or by carefully pipetting PFAS liquid (GENX and PFBS) into MilliQ water. To minimize adsorption losses to container walls, all experiments were conducted using polypropylene or polyethylene vessels. Two types of adsorption experiments were performed: kinetic studies and equilibrium adsorption tests.

For the kinetic studies, 50 mL of PFAS solution was transferred into a 50 mL polypropylene centrifuge tube, followed by the addition of 10 mg of the synthesized covalent organic framework (COF). The tubes were placed on an orbital shaker operating at 400 rpm to ensure uniform mixing. At predetermined time intervals, 2 mL samples were withdrawn using a polyethylene syringe for subsequent analysis. Equilibrium adsorption experiments were conducted by placing 25 mL of PFAS solution in a 50 mL polypropylene centrifuge tube with 10 mg of COF.

After adsorption, the samples were filtered through a 0.22 μm polypropylene filter to remove suspended solids. PFAS concentrations in the filtrates were quantified before and after adsorption using ultra-high-performance liquid chromatography coupled with mass spectrometry (UHPLC-MS) to get the adsorption data.

### S3.11 UHPLC MS/MS Analysis

Chromatographic separation was achieved using an Agilent ZORBAX RRHD Eclipse Plus C18 column (3 × 100 mm, 1.8 μm) column installed on an Agilent 1290 Infinity II UHPLC system. A gradient elution was performed using a mobile phase of 5 mM ammonium acetate in water (mobile phase A) and methanol (mobile phase B) at a flow rate of 0.4 mL/min for a total run time of 17 minutes with an injection volume of 2 μL. To minimize background PFAS contamination, the Agilent UHPLC system was equipped with a PFC-Free HPLC conversion kit and a PFC delay column to retain potential per- or polyfluorochemical impurities from the mobile phases.

Dynamic multiple reaction monitoring (dMRM) analysis was performed using an Agilent 6495C LC/TQ mass spectrometer with an Agilent jet stream (AJS) ion source operated in negative ionization mode. Data acquisition and processing were conducted using Agilent MassHunter data LC/MS Acquisition software version 10.0 and Quantitative Analysis software version 10.2, respectively. To enable quantification, calibration curves were prepared using the standard PFAC30PAR from Wellington.

### S3.12 SAXS Analysis

SAXS measurements were performed on the mySpot beamline (Zizak, 2016) of BESSY-II (Helmholtz-Zentrum Berlin, HZB, Germany) using a monochromatic X-ray beam at 18.0 keV aligned with a sample holder. SAXS data were collected with a large-area Dectris Eiger 9M detector as a single continuous signal. Small amounts of each powder sample (of 3 COFs) were deposited onto a Katpon tape. Each of the 3 samples was measured 32 times across a spatial coordinate grid 5 mm x 2 mm. The exposure time per frame (or point) was 10 seconds for all three samples, Each dataset was averaged, TAPB_DFTA_COF: 28 profiles averaged (4 rejected), TAPB_TA_COF: 10 profiles averaged (22 rejected), TAPB_DMTA_COF: 27 profiles averaged (5 rejected). Spurious and low-intensity profiles (due to e.g. heterogeneity in deposited powders) were filtered using a threshold of 5% of the maximum integrated intensity observed for that specific sample. Profiles with an integrated intensity below this value were rejected. The sample-to-detector distance of ∼330 mm allowed for a usable q-range of $\sim 0.07 < q < 38$ nm-1 . Transmission through the sample was calculated using an X-ray fluorescence signal collected from a lead beamstop using a RAYSPEC Sirius SD-E65133-BE-INC detector equipped with an 8 μm beryllium window,

where the incoming beam intensity was monitored and normalised using an ion chamber. The scattering q-range was calibrated against silicon (NIST SRM640) and the corresponding measured intensity was calibrated to absolute units against and a glassy carbon standard (NIST SRM3600, 1 mm, at T = 20 ℃). The resulting data were processed with custom Python scripts utilizing a pyfai library (Ashiotis et. al., J. Appl. Crystallogr. 48, 510–519, 2015. https://doi.org/10.1107/S1600576715004306). The processing steps included angle-dependent integration to 1D scattering curves and subtraction of an instrumental background (i.e. an "empty beamline" or "air" background) and the background of the Kaptoin tape.

## S4. Characterization of COFs

### S4.1 FTIR Analysis

FT-IR confirmed the successful formation of all three covalent organic frameworks through imine condensation. This is evidenced by the disappearance of characteristic vibrational bands of the starting monomers and the emergence of new bands corresponding to imine (C=N) linkages.

For TAPB-TA COF, the spectra of the pristine monomers, in figure S1 a and b, display the characteristic N-H stretching vibrations of TAPB at 3343 cm$^{-1}$ and 3430 cm⁻$^{1}$, as well as the carbonyl (C=O) stretching vibration of the TA linker at 1682 cm⁻$^{1}$. Following COF formation, these bands are no longer observed in the spectrum of the TAPB-TA COF, indicating consumption of the amine and aldehyde functionalities. Instead, a new band appears at 1618 cm⁻$^{1}$, which is assigned to the stretching vibration of the imine (C=N) bond, confirming successful Schiff base condensation.

A similar trend is observed for the TAPB-DFTA COF in figure S1 c and d. The DFTA linker exhibits a prominent C=O stretching band along with characteristic C-F vibrations at 543, 1017, and 1299 cm$^{-1}$. In the resulting COF, the N-H and C=O bands associated with the TAPB and DFTA monomers are absent, while a distinct imine stretching band emerges at 1620 cm$^{-1}$. Additionally, the C-F vibrational bands are retained but show slight shifts in position, consistent with incorporation of the fluorinated linker.

Likewise, the formation of the TAPB-DMTA COF is confirmed by the appearance of the imine stretching vibration, which is absent in the spectra of the individual monomers (Figure S1 e and f). The disappearance of the amine and carbonyl bands further confirms completion of the condensation reaction. Additional bands, such as the peak at 1442 cm$^{-1}$, can be attributed to the methyl ($CH_3$) groups of the DMTA linker, providing further evidence of its successful incorporation into the COF structure.

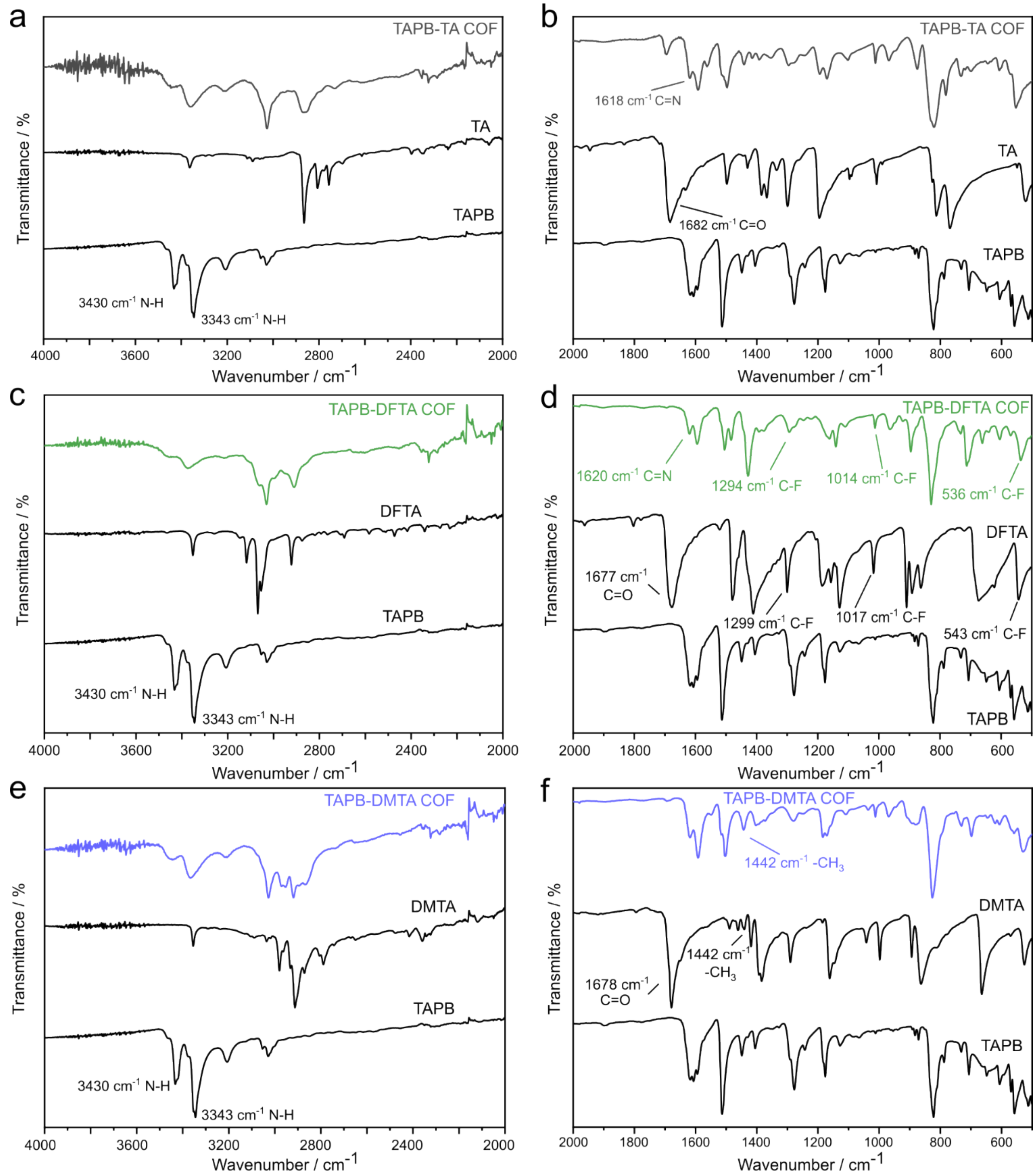


Figure S1 (a) FTIR spectra of TAPB-TA COF along with the linkers TAPB and TA in the regions 4000-2000 $cm^{-1}$ and (b) in the region 2000-500 $cm^{-1}$. (c) FTIR spectra of TAPB-DFTA COF along with the linkers TAPB and DFTA in the regions 4000-2000 $cm^{-1}$ and (d) in the region 2000-500 $cm^{-1}$. (e) FTIR spectra of TAPB-DMTA COF along with the linkers TAPB and DMTA in the regions 4000-2000 $cm^{-1}$ and (f) in the region 2000-500 $cm^{-1}$.

## S4.2 XPS analysis

XPS spectra confirming the formation of imine-linked COFs. All samples display a characteristic N 1s peak at 398.5 eV, corresponding to imine nitrogen, along with a small shoulder at higher binding energy attributed to surface amine species and minor surface oxidation. The DMTA-based COF shows a higher intensity C 1s peak at 284 eV, consistent with the presence of methyl substituents. The DFTA-based COF exhibits a characteristic F 1s peak at 689 eV, confirming the incorporation of fluorinated groups within the framework.

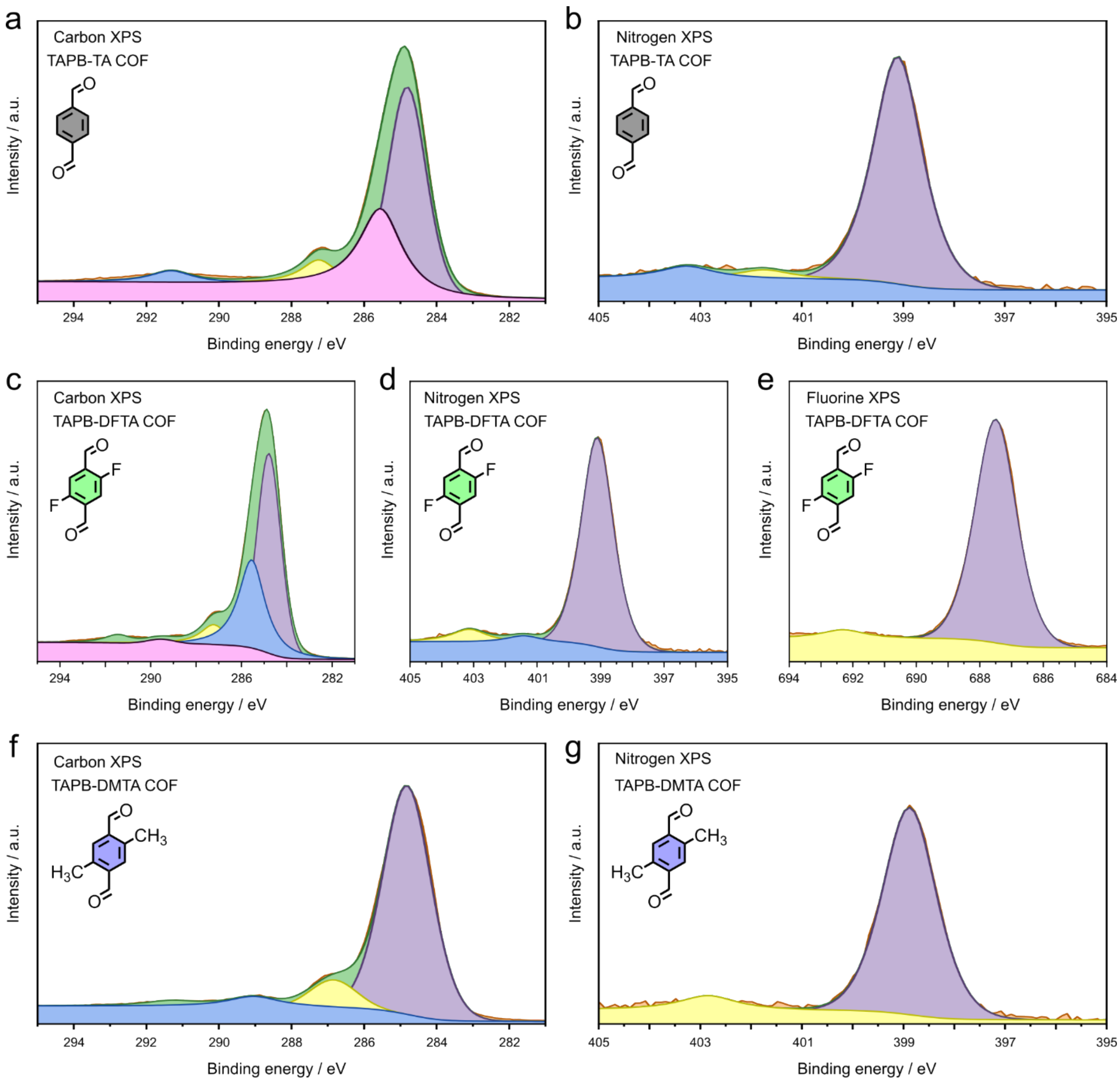

**Figure S2:** (a) Carbon XPS spectra and (b) nitrogen XPS spectra of TAPB-TA COF. (c) Carbon, (d) nitrogen and (e) fluorine XPS spectra of TAPB-DFTA COF. (f) Carbon and (g) nitrogen XPS spectra of TAPB-DMTA COF.

### S4.3 XRD analysis

Powder X-ray diffraction (XRD) analysis (Figure S3 a) reveals that all three COFs, namely TAPB-TA COF, TAPB-DFTA COF, and TAPB-DMTA COF exhibit closely similar diffraction patterns, indicative of comparable crystalline frameworks. Each COF displays a prominent low-angle reflection at $2\theta = 2.8°$, accompanied by weaker reflections at 4.9°, 5.6° and 7.5° which are characteristic of long range ordered porous structures.

As shown in Figures S3 b-d, the individual aldehyde and amine linkers exhibit distinct diffraction patterns prior to reaction. Upon COF formation, these linker specific reflections disappear, and a new, intense low angle diffraction peak emerges. The loss of the monomer diffraction features together with the appearance of this characteristic low-$2\theta$ reflection confirms the successful formation of crystalline COF structure for all three systems.

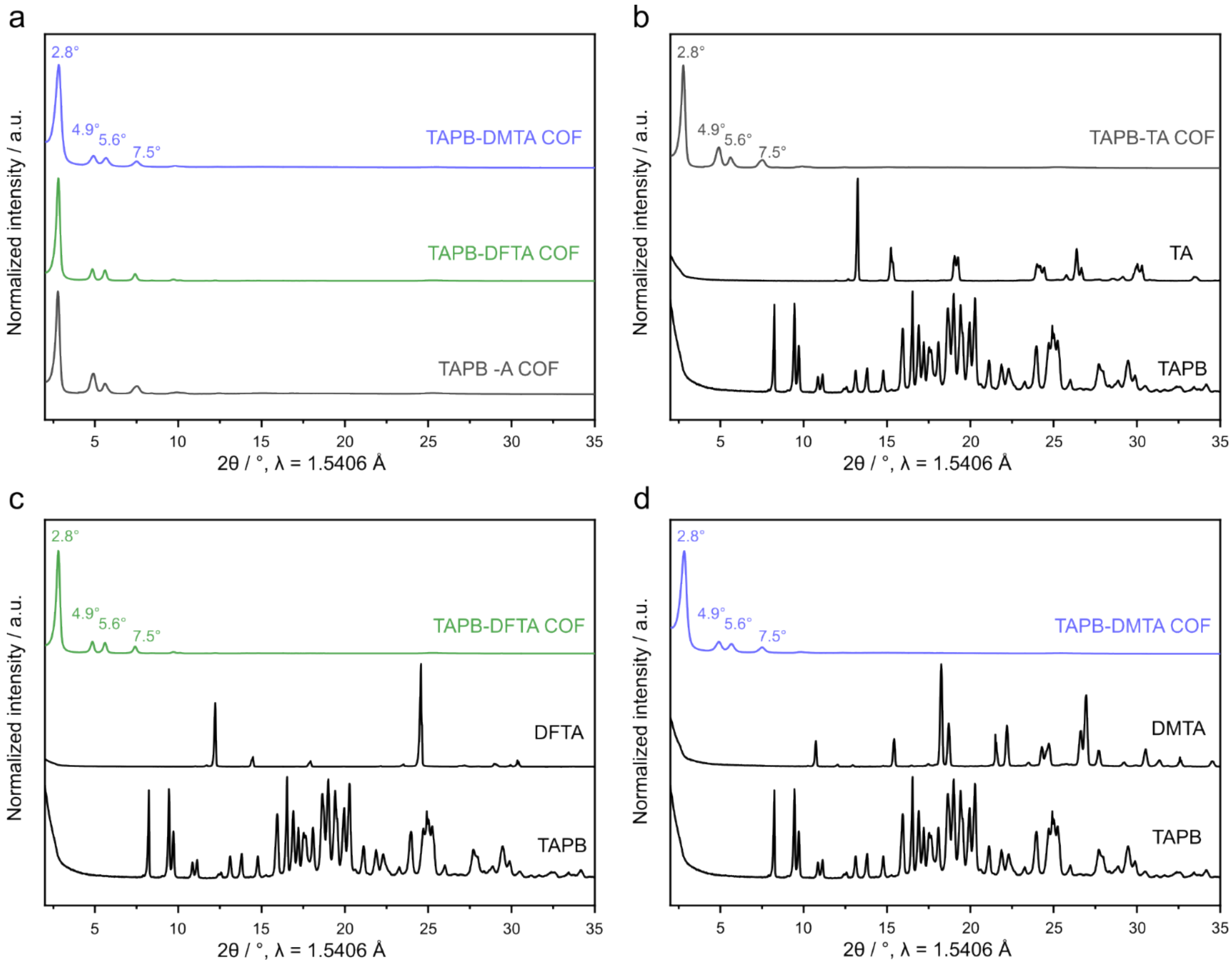


Figure S3 (a) Powder X-ray diffraction (XRD) patterns of the synthesized COFs, showing that all three COFs exhibit reflections at similar 2θ angles. (b) XRD patterns of TAPB-TA COF along with the individual linkers TAPB and TA. (c) XRD patterns of TAPB-DFTA COF along with the individual linkers TAPB and DFTA. (d) XRD patterns of TAPB-DMTA COF along with the individual linkers TAPB and DMTA.

## S4.4 Simulated structures

Materials Studio, a modelling and simulation software, was employed to simulate our COFs. The software offers an extensive suite of tools for visualising, constructing and analysing material structures. The software's user-friendly interface and advanced simulation capabilities enabled us to gain valuable insights into the structure of the COFs. Using Materials Studio, different stacking arrangements of the COF layers were systematically evaluated. Among the various possibilities, the AA stacking pattern was found to best match the experimental XRD data for all three COFs. Consequently, the simulated models of TAPB-TA COF, TAPB-DFTA COF and TAPB-DMTA COF were all described using AA stacked layers.

The unit cell parameters for each COF were determined as follows. For TAPB-TA COF, the space group is P□6 (174), with cell dimensions a = 35.5578 Å, b = 35.5578 Å, c = 3.5994 Å, and cell angles α = 90.0°, β = 90.0°, γ = 120.0°, yielding a unit cell volume of 3941.22 $Å^3$. The interlayer distance was calculated to be 3.6 Å (0.36 nm). For TAPB-DFTA COF, the space group is P□6(174), with cell dimensions a = 36.3822 Å, b = 36.3822 Å, c = 3.5402 Å, α = 90.0°, β = 90.0°, γ = 120.0°, and a unit cell volume of 4058.23 $Å^3$. The interlayer distance was determined to be 3.5 Å (0.35 nm). Finally, for TAPB-DMTA COF, the space group is P□6(174), with cell parameters a = 37.8177 Å, b = 37.8177 Å, c = 4.0798 Å, α = 90.0°, β = 90.0°, γ = 120.0°, resulting in a unit cell volume of 5053.12 $Å^3$. The interlayer distance was calculated to 4.1 Å (0.41 nm).

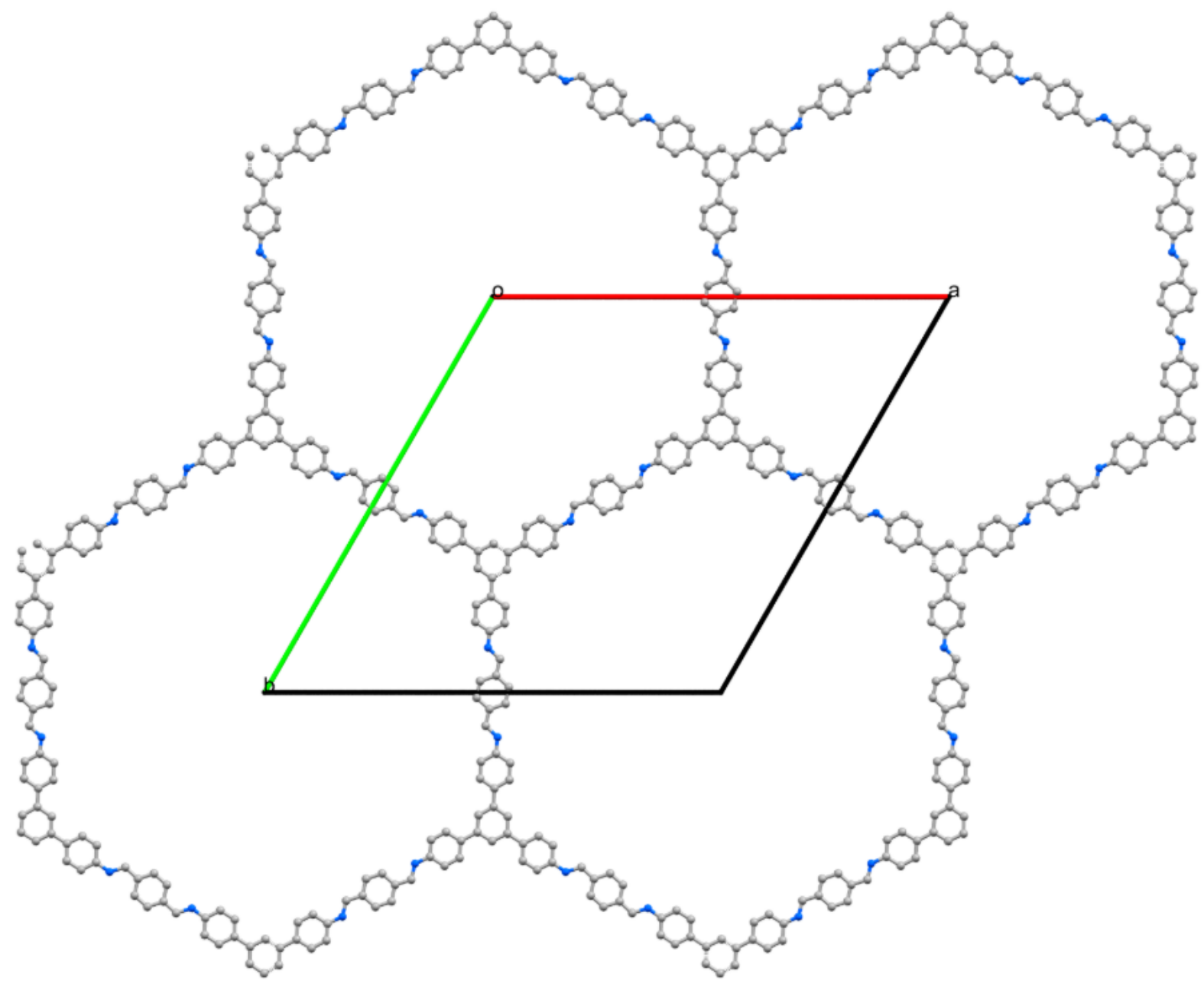

Figure S4: Simulated structure of TAPB-TA COF using Materials Studio.

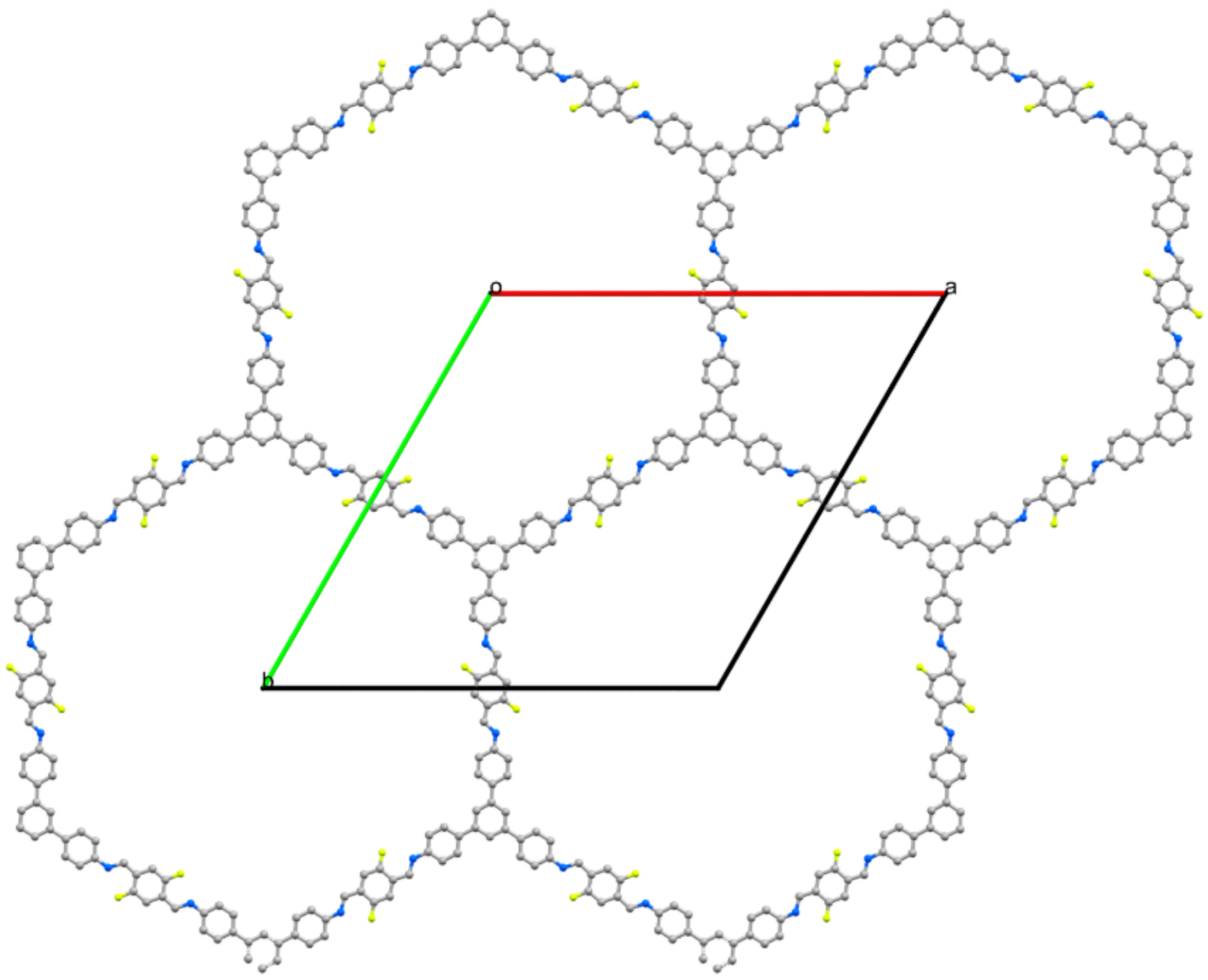


Figure S5: Simulated structure of TAPB-DFTA COF using Materials Studio.

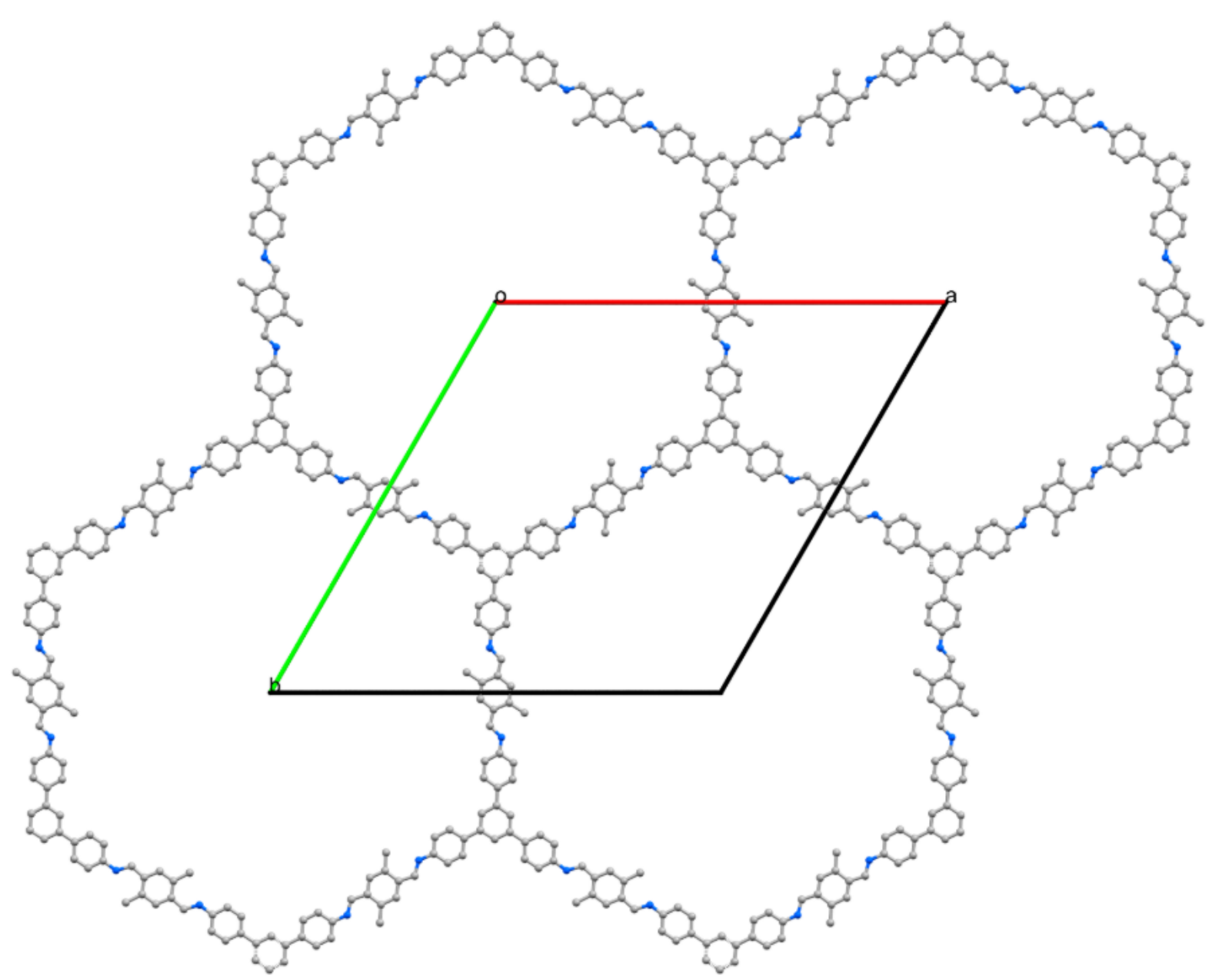


Figure S6: Simulated structure of TAPB-DMTA COF using Materials Studio.

### S4.5 Nitrogen adsorption

Table 1: Key parameters for the calculation of the BET specific surface area of TAPB-TA COF.

| Relative Pressure (p/p°) | 1/[Q(p°/p - 1)] |
|---|---|
| 0,00870 | 0,00121 |
| 0,01306 | 0,00156 |
| 0,01786 | 0,00195 |
| 0,02267 | 0,00227 |
| 0,02768 | 0,00260 |
| 0,03289 | 0,00295 |
| 0,03821 | 0,00331 |
| 0,04369 | 0,00369 |
| 0,04907 | 0,00405 |
| 0,06000 | 0,00480 |

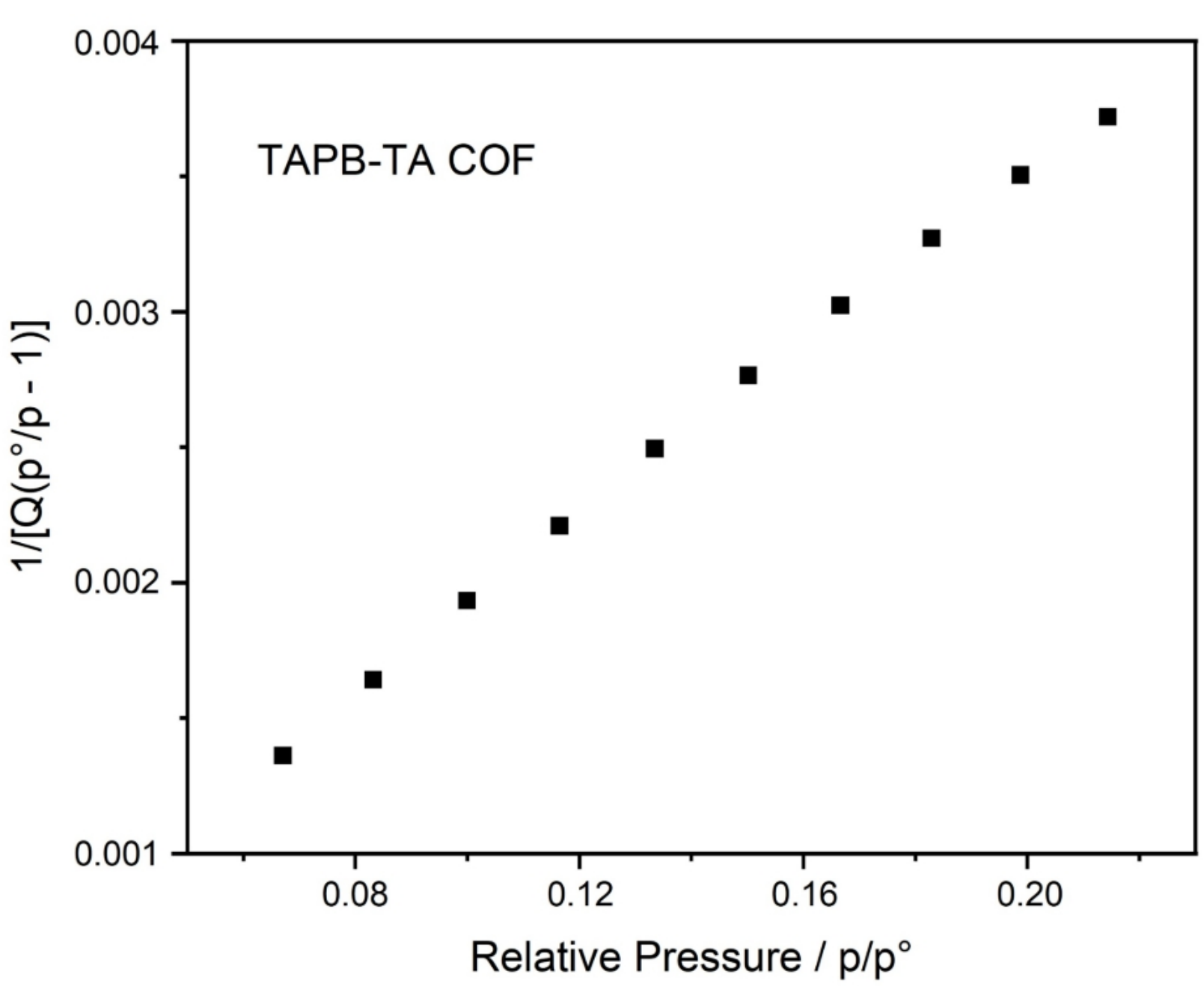

| | |
|---|---|
| 0,07091 | 0,00552 |

Figure S7: BET specific surface area plot of TAPB-TA COF.

Table 2: Key parameters for the calculation of the BET specific surface area of TAPB-DFTA COF.

| **Relative Pressure (p/p°)** | **1/[Q(p°/p - 1)]** |
|---|---|
| 0,02088 | 0,00010 |
| 0,02239 | 0,00010 |
| 0,02398 | 0,00011 |
| 0,02565 | 0,00011 |
| 0,02738 | 0,00012 |
| 0,02922 | 0,00012 |
| 0,03111 | 0,00013 |
| 0,03306 | 0,00014 |
| 0,03509 | 0,00015 |
| 0,03720 | 0,00015 |

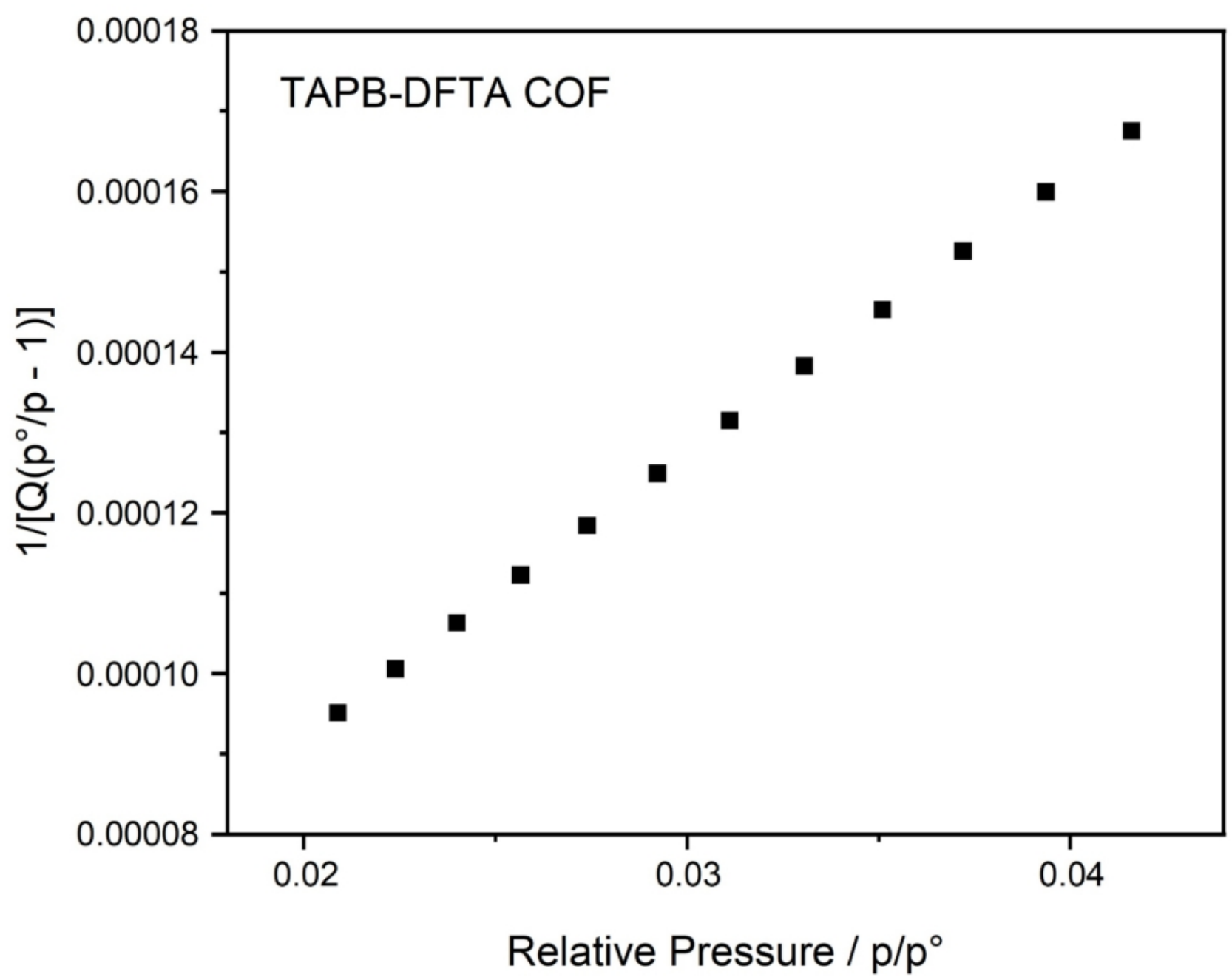

| | |
|---|---|
| 0,03936 | 0,00016 |
| 0,04159 | 0,00017 |

Figure S8: BET specific surface area plot of TAPB-DFTA COF

Table 3: Key parameters for the calculation of the BET specific surface area of TAPB-DMTA COF.

| Relative Pressure (p/p°) | 1/[Q(p°/p - 1)] |
|---|---|
| 0,00436 | 0,00006 |
| 0,00530 | 0,00007 |
| 0,00635 | 0,00008 |
| 0,00744 | 0,00009 |
| 0,00875 | 0,00010 |
| 0,01015 | 0,00012 |
| 0,01171 | 0,00013 |
| 0,01345 | 0,00014 |
| 0,01527 | 0,00016 |
| 0,01722 | 0,00017 |
| 0,01935 | 0,00019 |
| 0,02160 | 0,00020 |
| 0,02409 | 0,00022 |
| 0,02664 | 0,00024 |
| 0,02941 | 0,00026 |
| 0,03227 | 0,00028 |
| 0,03521 | 0,00030 |
| 0,03824 | 0,00032 |
| 0,04145 | 0,00034 |

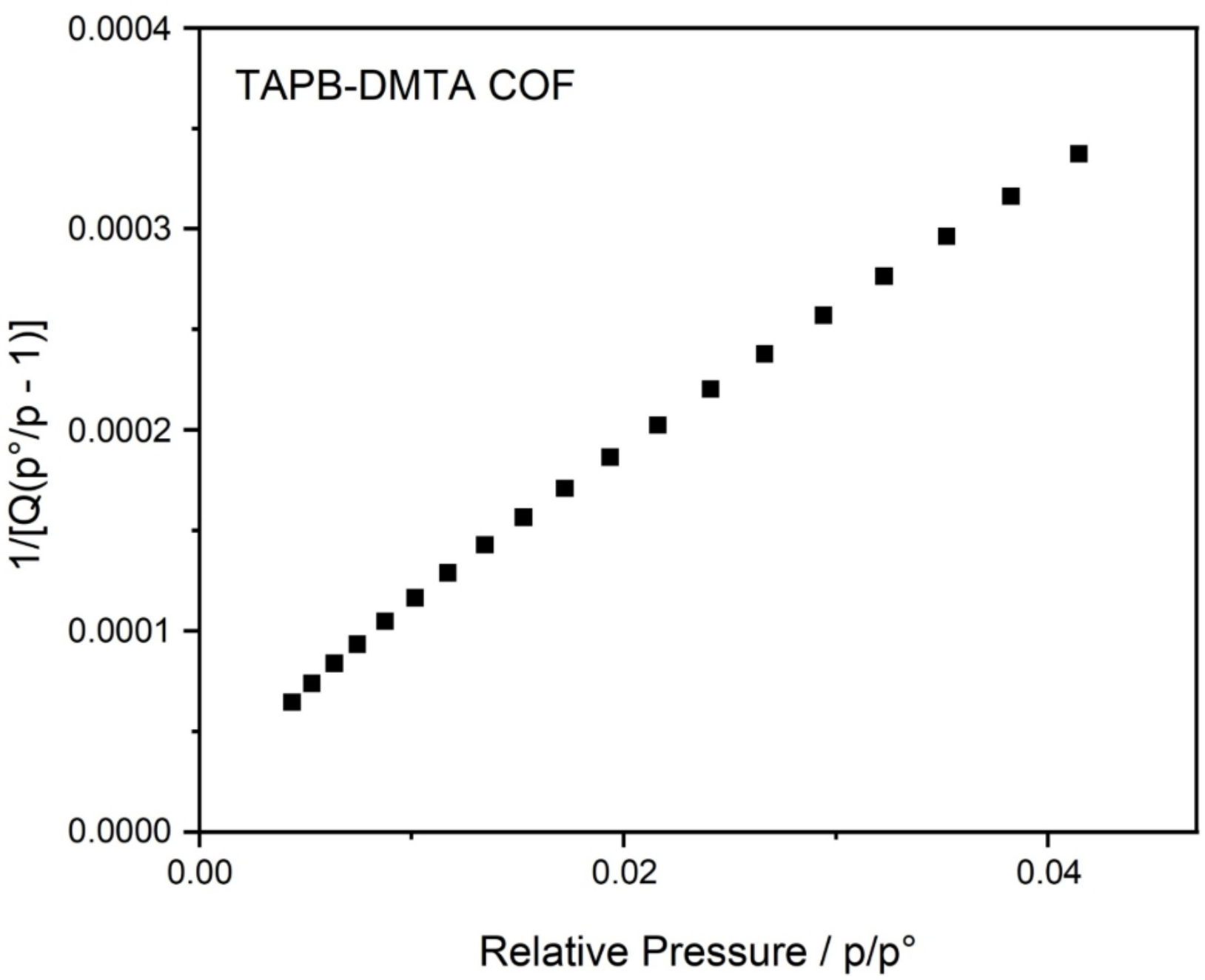


Figure S9: BET specific surface area plot of TAPB-DMTA COF.

Table 3: Key parameters for the calculation of the BET specific surface area of TAPB-DMTA COF.

| Sample | BET Surface Area ($m^2/g$) | C value | Qm ($cm^3/g$ STP) | Correlation coefficient | Molecular cross-sectional area ($nm^2$) |
|---|---|---|---|---|---|
| TAPB-TA COF | 262.0047 ± 2.6954 | 56.3459 | 60.1953 | 0.9996 | 0.1620 |
| TAPB-DFTA COF | 1237.0100 ± 3.7463 | 156.3790 | 284.2017 | 0.9999 | 0.1620 |
| TAPB-DMTA COF | 592.5120 ± 6.5708 | 179.8245 | 136.1290 | 0.9989 | 0.1620 |

### S4.6 TRIS XRD analysis

TRIS XRD was employed to monitor the imine condensation reaction between TAPB and TA, leading to the formation of TAPB-TA COF under mechanochemical conditions. The initial reaction was conducted by milling 105 mg of TAPB with 40 mg of TA in a transparent 14 mL polymethyl methacrylate (PMMA) jar, with 150 $\mu$L of mesitylene, 150 $\mu$L of 1,4-dioxane and 150 $\mu$L of glacial acetic acid as liquid additives. Two 5 mm stainless steel balls were used as the grinding medium, operating at a frequency of 50 Hz. As seen in figure S10, the COF started to form immediately and the reaction was stopped after 5 minutes as we did not obtain any information about the COF formation. Glacial acetic acid was diluted to 6M for future reactions to slow down COF formation enough to be able to see the reaction in TRIS XRD.

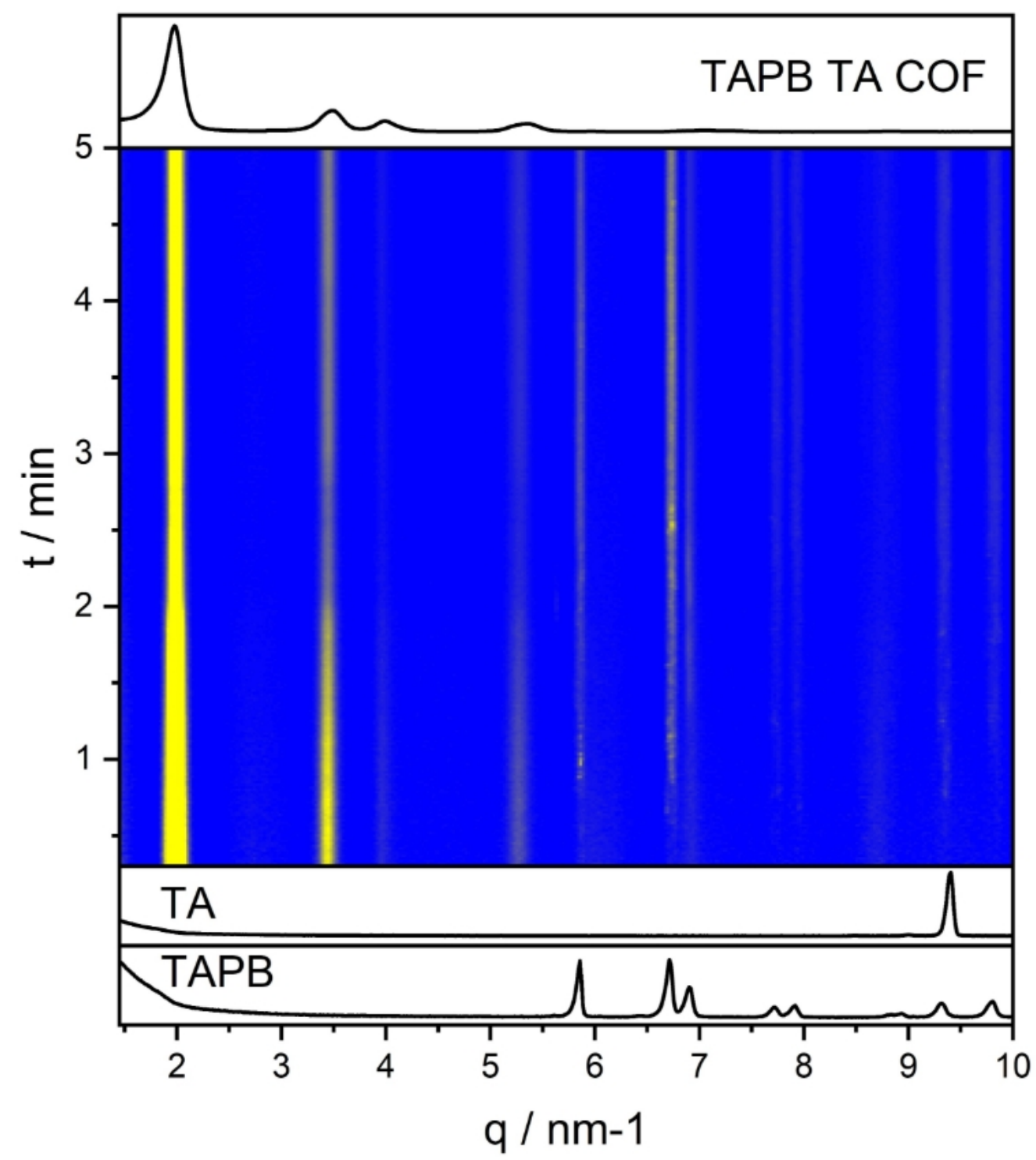


Figure S10: TRIS XRD pattern of TAPB-TA COF where the COF formation occurred instantly. The reaction was stopped after 5 minutes as no information was obtained about the formation process.

## S4.7 Stability tests

Stability tests were conducted for the three COFs under various conditions, including highly acidic and highly basic environments, as well as in solutions containing different concentrations of PFOA and PFOS. The results indicate that all three COFs remain structurally stable under these conditions and retain most of their crystallinity. However, a reduction in crystallinity was observed for all COFs after exposure to the pH 1 solution, suggesting

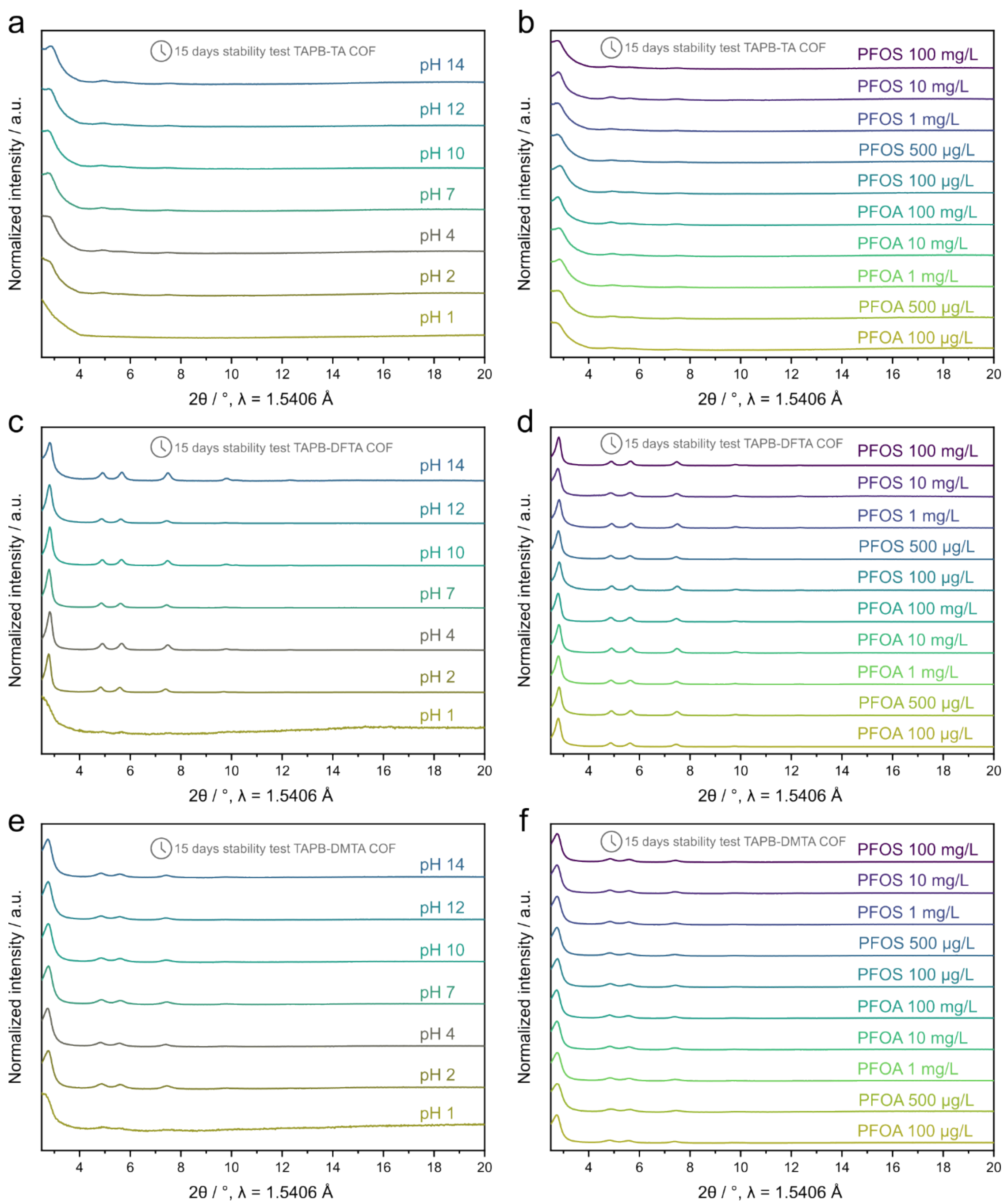


partial framework degradation under strongly acidic conditions.

Figure S11: Stability test of TAPB-TA COF in (a) different pH and (b) different PFAS solutions. Stability test of TAPB-DFTA COF in (c) different pH and (d) different PFAS solutions. Stability test of TAPB-DMTA COF in (e) different pH and (f) different PFAS solutions.

## S4.8 PFAS adsorption data

The kinetic PFAS adsorption data were fitted using several commonly applied kinetic models in order to identify the model that best describes the adsorption behavior. The models evaluated included the pseudo first order model, pseudo second order model, Elovich nonlinear model and the intraparticle diffusion model. Among the four models tested for fitting kinetic PFOA adsorption data using TAPB TA COF, the pseudo second order model provided the best agreement with the experimental data, yielding an $R^2$ value of 0.99999. In comparison, the pseudo first order, Elovich and intraparticle diffusion models produced lower $R^2$ values of 0.99961, 0.99945 and 0.77669 respectively. These results indicate that the pseudo second order model most accurately captures the adsorption kinetics for this system. The pseudo second order model fit suggests that the adsorption process is primarily governed by chemisorption, in which adsorption occurs through valence forces such as electron sharing or exchange between the PFAS molecules and the active sites of the COF surface. This implies that the adsorption rate is dependent on the availability of adsorption sites rather than solely on PFAS concentration in solution. Based

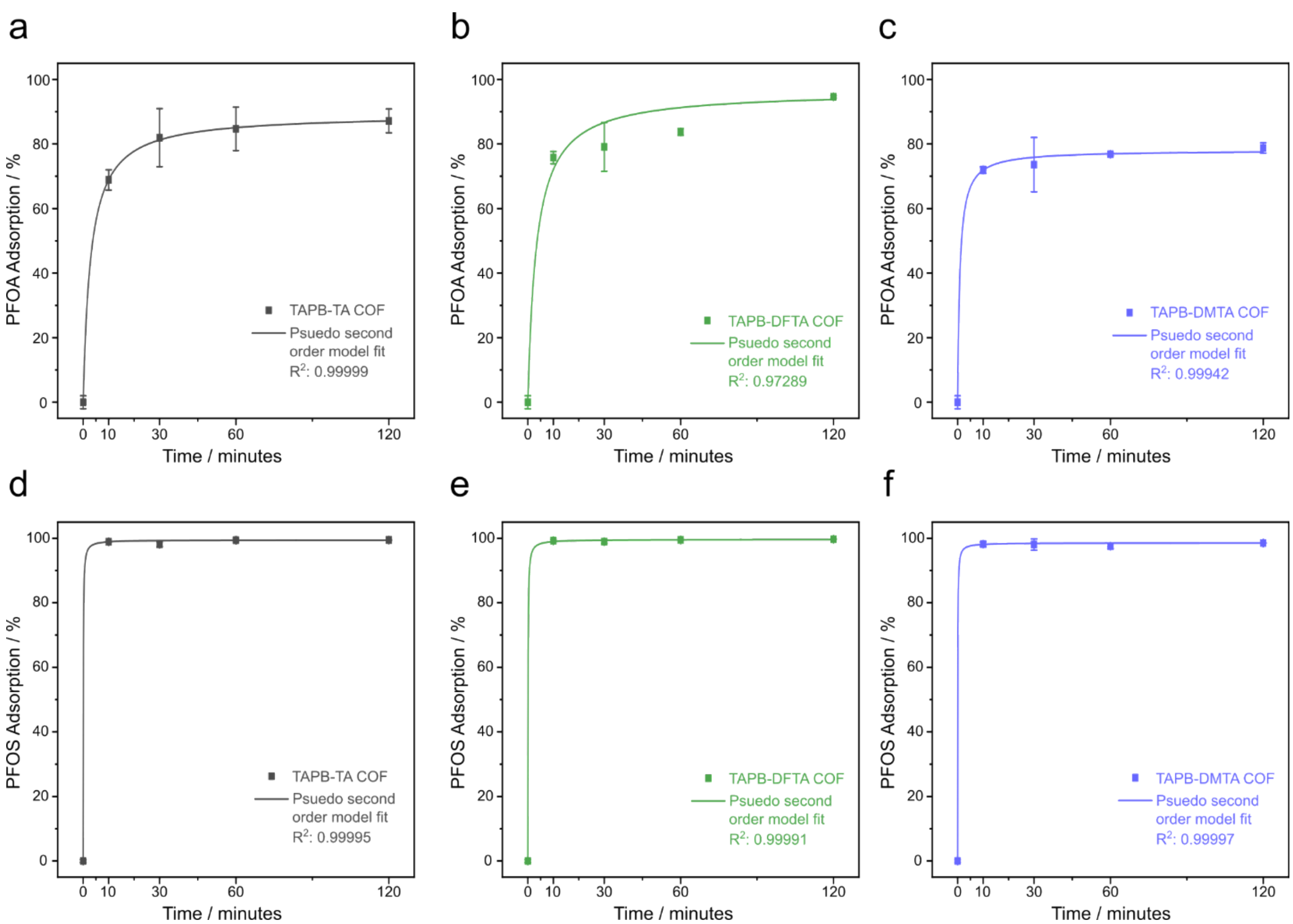

on comparison of the $R^2$ values, the pseudo second order model was also found to provide the best fit for the kinetic data of the remaining PFAS adsorption experiments and was therefore used to describe the kinetic behaviour throughout this study.

Figure S12: Psuedo second order model fit of PFOA kinetic adsorption data using (a) TAPB-TA COF (b) TAPB-DFTA COF (c) TAPB-DMTA COF. Psuedo second order model fit of PFOS kinetic adsorption data using (d) TAPB-TA COF (e) TAPB-DFTA COF (f) TAPB-DMTA COF.

Table 4: Method validation parameters for UHPLC-MS/MS analysis of PFAS

| Analyte | Detection Limit [pg/g] | Detection threshold [pg/g] | Determination limit[pg/g] | Correlation coefficient |
|---|---|---|---|---|
| PFOA | 0.2577062332368720 | 0.515412466473745 | 0.845872253116851 | 0.9996 |
| PFOS | 0.41870103033204 | 0.837402060664079 | 1.33770295370883 | 0.9989 |
| GENX | 0.750426228993702 | 1.50085245579874 | 2.30053394320473 | 0.9968 |
| PFBS | 0.270752212573656 | 0.541504425147311 | 0.882112764501027 | 0.9994 |

| Name | Precursor Ion | MS1 Res | Prodcut Ion | MS2 Res | Ret time (min) | Delta Ret time | Fragmentor | Collision energy | Cell accelerator voltage | Polarity |
|---|---|---|---|---|---|---|---|---|---|---|
| PFOA | 413 | Unit | 369 | Unit | 7.97 | 1.99 | 166 | 7 | 2 | Negative |
| PFOA | 413 | Unit | 169 | Unit | 7.97 | 1.99 | 166 | 19 | 2 | Negative |
| PFOS | 498.9 | Unit | 99 | Unit | 8.83 | 2.21 | 166 | 48 | 4 | Negative |
| PFOS | 498.9 | Unit | 80 | Unit | 8.83 | 2.21 | 166 | 52 | 4 | Negative |
| GENX | 285 | Unit | 185 | Unit | 6.27 | 1.57 | 166 | 19 | 5 | Negative |
| GENX | 285 | Unit | 169 | Unit | 6.27 | 1.57 | 166 | 3 | 5 | Negative |
| PFBS | 298.9 | Unit | 99 | Unit | 5.13 | 1.28 | 166 | 36 | 2 | Negative |
| PFBS | 298.9 | Unit | 80 | Unit | 5.13 | 1.28 | 166 | 43 | 2 | Negative |
| 13C8 PFOA | 421 | Unit | 376 | Unit | 7.97 | 1.99 | 166 | 7 | 2 | Negative |

| | | | | | | | | | | |
|---|---|---|---|---|---|---|---|---|---|---|
| 13C8 PFOS | 507 | Unit | 80 | Unit | 8.83 | 2.21 | 166 | 52 | 4 | Negative |
| 13C3 PFBS | 302 | Unit | 80 | Unit | 5.14 | 1.29 | 166 | 43 | 2 | Negative |

Table 5: MS/MS transitions parameters for PFAS determination.

The stated PFAS concentrations (e.g., 100, 10, 1 mg L⁻$^{1}$, 500 μg/L and 100 μg/L) refer to the target concentrations that we aimed for during sample preparation. Subsequent UHPLC-MS/MS analysis showed that the actual concentrations deviated slightly from these nominal values. These deviations are attributed primarily to the hygroscopic nature of some PFAS standards, which made accurate weighing challenging despite careful handling. Since the objective of the adsorption experiments was to investigate adsorption behavior over a range of concentrations rather than at exact nominal values, the measured concentrations were used for all subsequent calculations and data analysis. Secondly, adsorption efficiencies and relative concentration changes in percentage were calculated using the experimentally determined initial concentration (C₀) for each sample, which was normalized to 100% and all remaining concentrations were expressed relative to this value.

Table 6: Kinetic study of adsorption of 100 μg/L PFOA

| **Serial number** | **Measurement** | **Amount μg/L** | **Error μg/L** | **Percentage** | **Error** |
|---|---|---|---|---|---|
| 1 | PFOA μg/L | 75,05671276 | 1,514543997 | 100,0 | 2,0 |
| 2 | TAPB TA COF 10 minute | 23,3735512 | 2,364693322 | 31,1 | 3,2 |
| 3 | TAPB TA COF 30 minutes | 13,54825664 | 6,771257429 | 18,1 | 9,0 |
| 4 | TAPB TA COF 60 minutes | 11,50319813 | 5,042356112 | 15,3 | 6,7 |
| 5 | TAPB DFTA COF 120 minutes | 9,648470775 | 2,760153824 | 12,9 | 3,7 |
| 6 | TAPB DFTA COF 10 minute | 18,18493775 | 1,424180016 | 24,2 | 1,9 |

| | | | | | |
|---|---|---|---|---|---|
| 7 | TAPB DFTA COF 30 minutes | 15,70583935 | 5,66431014 | 20,9 | 7,5 |
| 8 | TAPB DFTA COF 60 minutes | 12,20337834 | 0,794637696 | 16,3 | 1,1 |
| 9 | TAPB DFTA COF 120 minutes | 4,03484436 | 0,284988124 | 5,4 | 0,4 |
| 10 | TAPB DMTA COF 10 minute | 21,05165536 | 0,792018563 | 28,0 | 1,1 |
| 11 | TAPB DMTA COF 30 minutes | 19,82545668 | 6,304491132 | 26,4 | 8,4 |
| 12 | TAPB DMTA COF 60 minutes | 17,39990123 | 0,37388211 | 23,2 | 0,5 |
| 13 | TAPB DMTA COF 120 minutes | 15,91707542 | 1,210697949 | 21,2 | 1,6 |

Table 7: Kinetic study of adsorption of 100 μg/L PFOS

| Serial number | Measurement | Amount μg/L | Error μg/L | Percentage | Error |
|---|---|---|---|---|---|
| 1 | PFOS μg/L | 24,47058385 | 0,109223571 | 100,0 | 0,4 |
| 2 | TAPB TA COF 10 minute | 0,271381607 | 0,033728482 | 1,1 | 0,1 |
| 3 | TAPB TA COF 30 minutes | 0,469818802 | 0,18364975 | 1,9 | 0,8 |
| 4 | TAPB TA COF 60 minutes | 0,151574786 | 0,130927319 | 0,6 | 0,5 |
| 5 | TAPB DFTA COF 120 minutes | 0,139648532 | 0,028219639 | 0,6 | 0,1 |
| 6 | TAPB DFTA COF 10 minute | 0,193054569 | 0,082928782 | 0,8 | 0,3 |
| 7 | TAPB DFTA COF 30 minutes | 0,264944829 | 0,136351988 | 1,1 | 0,6 |
| 8 | TAPB DFTA COF 60 minutes | 0,131941036 | 0,011137356 | 0,5 | 0,0 |
| 9 | TAPB DFTA COF 120 minutes | 0,077669448 | 0,019809322 | 0,3 | 0,1 |
| 10 | TAPB DMTA COF 10 minute | 0,45353539 | 0,170545964 | 1,9 | 0,7 |
| 11 | TAPB DMTA COF 30 minutes | 0,481787365 | 0,428669557 | 2,0 | 1,8 |
| 12 | TAPB DMTA COF 60 minutes | 0,605206268 | 0,19934468 | 2,5 | 0,8 |

| | | | | | |
|---|---|---|---|---|---|
| 13 | TAPB DMTA COF 120 minutes | 0,367788417 | 0,018753596 | 1,5 | 0,1 |

Table 8: Kinetic study of adsorption of 100 μg/L GENX

| Serial number | Measurement | Amount μg/L | Error μg/L | Percentage | Error |
|---|---|---|---|---|---|
| 1 | GENX 100 μg/L | 8,61069184 | 0,086190635 | 100,0 | 1,0 |
| 2 | TAPB TA COF 10 minute | 4,84532722 | 1,393920282 | 56,3 | 16,2 |
| 3 | TAPB TA COF 30 minutes | 5,06133963 | 0,54068564 | 58,8 | 6,3 |
| 4 | TAPB TA COF 60 minutes | 5,613989699 | 0,298322299 | 65,2 | 3,5 |
| 5 | TAPB DFTA COF 120 minutes | 5,259168463 | 0,155193815 | 61,1 | 1,8 |
| 6 | TAPB DFTA COF 10 minute | 4,992435879 | 0,101661797 | 58,0 | 1,2 |
| 7 | TAPB DFTA COF 30 minutes | 5,23674426 | 0,464594576 | 60,8 | 5,4 |
| 8 | TAPB DFTA COF 60 minutes | 4,607054829 | 0,095791664 | 53,5 | 1,1 |
| 9 | TAPB DFTA COF 120 minutes | 3,918551889 | 0,252677254 | 45,5 | 2,9 |
| 10 | TAPB DMTA COF 10 minute | 6,107255852 | 0,208715617 | 70,9 | 2,4 |
| 11 | TAPB DMTA COF 30 minutes | 5,284492311 | 0,500119812 | 61,4 | 5,8 |
| 12 | TAPB DMTA COF 60 minutes | 5,716725658 | 0,184321771 | 66,4 | 2,1 |
| 13 | TAPB DMTA COF 120 minutes | 5,891123672 | 0,09958944 | 68,4 | 1,2 |

Table 9: Kinetic study of adsorption of 100 μg/L PFBS

| Serial number | Measurement | Amount μg/L | Error μg/L | Percentage | Error |
|---|---|---|---|---|---|
| 1 | PFBS 100 μg/L | 23,66401212 | 0,08943707 | 100,0 | 0,4 |

| | | | | | |
|---|---|---|---|---|---|
| 2 | TAPB TA COF 10 minute | 18,83121603 | 0,578506411 | 79,6 | 2,4 |
| 3 | TAPB TA COF 30 minutes | 17,98454012 | 0,474884217 | 76,0 | 2,0 |
| 4 | TAPB TA COF 60 minutes | 18,32213768 | 0,607370869 | 77,4 | 2,6 |
| 5 | TAPB DFTA COF 120 minutes | 17,63068157 | 0,179268687 | 74,5 | 0,8 |
| 6 | TAPB DFTA COF 10 minute | 19,59973234 | 0,837381603 | 82,8 | 3,5 |
| 7 | TAPB DFTA COF 30 minutes | 18,68698937 | 0,107435149 | 79,0 | 0,5 |
| 8 | TAPB DFTA COF 60 minutes | 17,44478274 | 0,700424732 | 73,7 | 3,0 |
| 9 | TAPB DFTA COF 120 minutes | 13,30919922 | 0,034037111 | 56,2 | 0,1 |
| 10 | TAPB DMTA COF 10 minute | 20,26397504 | 0,273348099 | 85,6 | 1,2 |
| 11 | TAPB DMTA COF 30 minutes | 20,74989983 | 0,870157567 | 87,7 | 3,7 |
| 12 | TAPB DMTA COF 60 minutes | 20,86586275 | 0,126179376 | 88,2 | 0,5 |
| 13 | TAPB DMTA COF 120 minutes | 20,25144604 | 0,473776543 | 85,6 | 2,0 |

Table 10: Study of adsorption using different concentrations of PFOA

| Serial number | Measurement | Amount mg/L | Error mg/L | Percentage | Error |
|---|---|---|---|---|---|
| 1 | PFOA 100 mg/L | 78,88682314 | 8,859754312 | 100,0 | 11,2 |
| 2 | TAPB TA COF 24 hours | 65,02082649 | 1,653877807 | 82,4 | 2,1 |
| 3 | PFOA 10 mg/L | 7,686800602 | 0,72604027 | 100,0 | 9,4 |
| 4 | TAPB TA COF 24 hours | 7,279400555 | 0,019749299 | 94,7 | 0,3 |
| 5 | PFOA 1 mg/L | 0,734231688 | 0,01003475 | 100,0 | 1,4 |
| 6 | TAPB TA COF 24 hours | 0,671257165 | 0,053965258 | 91,4 | 7,3 |
| 7 | PFOA 500 μg/L | 0,377490299 | 0,014128163 | 100,0 | 3,7 |
| 8 | TAPB TA COF 24 hours | 0,262179241 | 0,019773331 | 69,5 | 5,2 |

| | | | | | |
|---|---|---|---|---|---|
| 9 | PFOA 100 μg/L | 0,075056713 | 0,001514544 | 100,0 | 2,0 |
| 10 | TAPB TA COF 24 hours | 0,029703783 | 0,003466078 | 39,6 | 4,6 |
| 11 | PFOA 100 mg/L | 78,88682314 | 8,859754312 | 100,0 | 11,2 |
| 12 | TAPB DFTA COF 24 hours | 40,97458934 | 5,548107229 | 51,9 | 7,0 |
| 13 | PFOA 10 mg/L | 7,686800602 | 0,72604027 | 100,0 | 9,4 |
| 14 | TAPB DFTA COF 24 hours | 4,034006019 | 0,078607206 | 52,5 | 1,0 |
| 15 | PFOA 1 mg/L | 0,734231688 | 0,01003475 | 100,0 | 1,4 |
| 16 | TAPB DFTA COF 24 hours | 0,336775693 | 0,023992112 | 45,9 | 3,3 |
| 17 | PFOA 500 μg/L | 0,377490299 | 0,014128163 | 100,0 | 3,7 |
| 18 | TAPB DFTA COF 24 hours | 0,125069899 | 0,018325774 | 33,1 | 4,9 |
| 19 | PFOA 100 μg/L | 0,075056713 | 0,001514544 | 100,0 | 2,0 |
| 20 | TAPB DFTA COF 24 hours | 0,009494772 | 0,000480526 | 12,7 | 0,6 |
| 21 | PFOA 100 mg/L | 78,88682314 | 8,859754312 | 100,0 | 11,2 |
| 22 | TAPB DMTA COF 24 hours | 73,78009757 | 1,358705054 | 93,5 | 1,7 |
| 23 | PFOA 10 mg/L | 7,686800602 | 0,72604027 | 100,0 | 9,4 |
| 24 | TAPB DMTA COF 24 hours | 6,69766307 | 0,395847239 | 87,1 | 5,1 |
| 25 | PFOA 1 mg/L | 0,734231688 | 0,01003475 | 100,0 | 1,4 |
| 26 | TAPB DMTA COF 24 hours | 0,690179211 | 0,047543065 | 94,0 | 6,5 |
| 27 | PFOA 500 μg/L | 0,377490299 | 0,014128163 | 100,0 | 3,7 |
| 28 | TAPB DMTA COF 24 hours | 0,359459101 | 0,01257321 | 95,2 | 3,3 |
| 29 | PFOA 100 μg/L | 0,075056713 | 0,001514544 | 100,0 | 2,0 |
| 30 | TAPB DMTA COF 24 hours | 0,040717134 | 0,002279296 | 54,2 | 3,0 |

Table 11: Study of adsorption using different concentrations of PFOS

| **Serial number** | **Measurement** | **Amount mg/L** | **Error mg/L** | **Percentage** | **Error** |
|---|---|---|---|---|---|
| 1 | PFOS 100 mg/L | 27,19777092 | 0,86219314 | 100,0 | 3,2 |

| | | | | | |
|---|---|---|---|---|---|
| 2 | TAPB TA COF 24 hours | 18,4872099 | 0,178672103 | 68,0 | 0,7 |
| 3 | PFOS 10 mg/L | 2,733767999 | 0,026749279 | 100,0 | 1,0 |
| 4 | TAPB TA COF 24 hours | 1,606104797 | 0,01761334 | 58,8 | 0,6 |
| 5 | PFOS 1 mg/L | 0,29618502 | 0,005185981 | 100,0 | 1,8 |
| 6 | TAPB TA COF 24 hours | 0,135928127 | 0,000349713 | 45,9 | 0,1 |
| 7 | PFOS 500 μg/L | 0,150055815 | 0,001756228 | 100,0 | 1,2 |
| 8 | TAPB TA COF 24 hours | 0,043787251 | 9,37309E-05 | 29,2 | 0,1 |
| 9 | PFOS 100 μg/L | 0,024470584 | 0,000109224 | 100,0 | 0,4 |
| 10 | TAPB TA COF 24 hours | 0,001943583 | 0,0003259 | 7,9 | 1,3 |
| 11 | PFOS 100 mg/L | 27,19777092 | 0,86219314 | 100,0 | 3,2 |
| 12 | TAPB DFTA COF 24 hours | 11,03570912 | 0,056937374 | 40,6 | 0,2 |
| 13 | PFOS 10 mg/L | 2,733767999 | 0,026749279 | 100,0 | 1,0 |
| 14 | TAPB DFTA COF 24 hours | 0,800491944 | 0,064592181 | 29,3 | 2,4 |
| 15 | PFOS 1 mg/L | 0,29618502 | 0,005185981 | 100,0 | 1,8 |
| 16 | TAPB DFTA COF 24 hours | 0,056026504 | 0,004606534 | 18,9 | 1,6 |
| 17 | PFOS 500 μg/L | 0,150055815 | 0,001756228 | 100,0 | 1,2 |
| 18 | TAPB DFTA COF 24 hours | 0,013151207 | 0,000194521 | 8,8 | 0,1 |
| 19 | PFOS 100 μg/L | 0,024470584 | 0,000109224 | 100,0 | 0,4 |
| 20 | TAPB DFTA COF 24 hours | 0,000511068 | 1,44597E-05 | 2,1 | 0,1 |
| 21 | PFOS 100 mg/L | 27,19777092 | 0,86219314 | 100,0 | 3,2 |
| 22 | TAPB DMTA COF 24 hours | 23,64915217 | 0,431495772 | 87,0 | 1,6 |
| 23 | PFOS 10 mg/L | 2,733767999 | 0,026749279 | 100,0 | 1,0 |
| 24 | TAPB DMTA COF 24 hours | 1,991632519 | 0,001380881 | 72,9 | 0,1 |
| 25 | PFOS 1 mg/L | 0,29618502 | 0,005185981 | 100,0 | 1,8 |
| 26 | TAPB DMTA COF 24 hours | 0,189110034 | 0,006296178 | 63,8 | 2,1 |
| 27 | PFOS 500 μg/L | 0,150055815 | 0,001756228 | 100,0 | 1,2 |
| 28 | TAPB DMTA COF 24 hours | 0,084519716 | 0,003716147 | 56,3 | 2,5 |
| 29 | PFOS 100 μg/L | 0,024470584 | 0,000109224 | 100,0 | 0,4 |

| | | | | | |
|---|---|---|---|---|---|
| 30 | TAPB DMTA COF 24 hours | 0,006440801 | 0,000151887 | 26,3 | 0,6 |

Table 12: Study of adsorption using different concentrations of GENX

| Serial number | Measurement | Amount mg/L | Error mg/L | Percentage | Error |
|---|---|---|---|---|---|
| 1 | GENX 100 mg/L | 8,045563994 | 0,211857293 | 100,0 | 2,6 |
| 2 | TAPB TA COF 24 hours | 6,823622564 | 0,08157715 | 84,8 | 1,0 |
| 3 | GENX 10 mg/L | 1,026722939 | 0,017368584 | 100,0 | 1,7 |
| 4 | TAPB TA COF 24 hours | 0,719308009 | 0,016112078 | 70,1 | 1,6 |
| 5 | GENX 1 mg/L | 0,079823642 | 0,001084156 | 100,0 | 1,4 |
| 6 | TAPB TA COF 24 hours | 0,069030727 | 0,001543561 | 86,5 | 1,9 |
| 7 | GENX 500 μg/L | 0,046851989 | 0,001240138 | 100,0 | 2,6 |
| 8 | TAPB TA COF 24 hours | 0,0363311 | 0,000306385 | 77,5 | 0,7 |
| 9 | GENX 100 μg/L | 0,008610692 | 8,61906E-05 | 100,0 | 1,0 |
| 10 | TAPB TA COF 24 hours | 0,006117759 | 0,000147366 | 71,0 | 1,7 |
| 11 | GENX 100 mg/L | 8,045563994 | 0,211857293 | 100,0 | 2,6 |
| 12 | TAPB DFTA COF 24 hours | 6,323530499 | 0,102882961 | 78,6 | 1,3 |
| 13 | GENX 10 mg/L | 1,026722939 | 0,017368584 | 100,0 | 1,7 |
| 14 | TAPB DFTA COF 24 hours | 0,648759769 | 0,008045483 | 63,2 | 0,8 |
| 15 | GENX 1 mg/L | 0,079823642 | 0,001084156 | 100,0 | 1,4 |
| 16 | TAPB DFTA COF 24 hours | 0,059459276 | 0,000923011 | 74,5 | 1,2 |
| 17 | GENX 500 μg/L | 0,046851989 | 0,001240138 | 100,0 | 2,6 |
| 18 | TAPB DFTA COF 24 hours | 0,028336458 | 0,000626834 | 60,5 | 1,3 |
| 19 | GENX 100 μg/L | 0,008610692 | 8,61906E-05 | 100,0 | 1,0 |
| 20 | TAPB DFTA COF 24 hours | 0,00413453 | 0,000131956 | 48,0 | 1,5 |
| 21 | GENX 100 mg/L | 8,045563994 | 0,211857293 | 100,0 | 2,6 |
| 22 | TAPB DMTA COF 24 hours | 7,053572581 | 0,190284012 | 87,7 | 2,4 |

| | | | | | |
|---|---|---|---|---|---|
| 23 | GENX 10 mg/L | 1,026722939 | 0,017368584 | 100,0 | 1,7 |
| 24 | TAPB DMTA COF 24 hours | 0,735921566 | 0,000258972 | 71,7 | 0,0 |
| 25 | GENX 1 mg/L | 0,079823642 | 0,001084156 | 100,0 | 1,4 |
| 26 | TAPB DMTA COF 24 hours | 0,071607524 | 0,000809463 | 89,7 | 1,0 |
| 27 | GENX 500 μg/L | 0,046851989 | 0,001240138 | 100,0 | 2,6 |
| 28 | TAPB DMTA COF 24 hours | 0,036173855 | 0,000794437 | 77,2 | 1,7 |
| 29 | GENX 100 μg/L | 0,008610692 | 8,61906E-05 | 100,0 | 1,0 |
| 30 | TAPB DMTA COF 24 hours | 0,006402996 | 0,000153275 | 74,4 | 1,8 |

Table 13: Study of adsorption using different concentrations of PFBS

| Serial number | Measurement | Amount mg/L | Error mg/L | Percentage | Error |
|---|---|---|---|---|---|
| 1 | PFBS 100 mg/L | 28,9579556 | 0,626835676 | 100,0 | 2,2 |
| 2 | TAPB TA COF 24 hours | 24,56916329 | 0,101597797 | 84,8 | 0,4 |
| 3 | PFBS 10 mg/L | 2,918136743 | 0,010359508 | 100,0 | 0,4 |
| 4 | TAPB TA COF 24 hours | 2,554733196 | 0,023024754 | 87,5 | 0,8 |
| 5 | PFBS 1 mg/L | 0,275851083 | 0,008121844 | 100,0 | 2,9 |
| 6 | TAPB TA COF 24 hours | 0,259525145 | 0,002328138 | 94,1 | 0,8 |
| 7 | PFBS 500 μg/L | 0,134318962 | 0,003426736 | 100,0 | 2,6 |
| 8 | TAPB TA COF 24 hours | 0,126539655 | 0,003049217 | 94,2 | 2,3 |
| 9 | PFBS 100 μg/L | 0,023664012 | 8,94371E-05 | 100,0 | 0,4 |
| 10 | TAPB TA COF 24 hours | 0,021677387 | 0,000482986 | 91,6 | 2,0 |
| 11 | PFBS 100 mg/L | 28,9579556 | 0,626835676 | 100,0 | 2,2 |
| 12 | TAPB DFTA COF 24 hours | 27,54458692 | 6,825143286 | 95,1 | 23,6 |
| 13 | PFBS 10 mg/L | 2,918136743 | 0,010359508 | 100,0 | 0,4 |
| 14 | TAPB DFTA COF 24 hours | 2,263207776 | 0,042939924 | 77,6 | 1,5 |
| 15 | PFBS 1 mg/L | 0,275851083 | 0,008121844 | 100,0 | 2,9 |

| | | | | | |
|---|---|---|---|---|---|
| 16 | TAPB DFTA COF 24 hours | 0,210776944 | 0,005107577 | 76,4 | 1,9 |
| 17 | PFBS 500 μg/L | 0,134318962 | 0,003426736 | 100,0 | 2,6 |
| 18 | TAPB DFTA COF 24 hours | 0,107426724 | 0,00177525 | 80,0 | 1,3 |
| 19 | PFBS 100 μg/L | 0,023664012 | 8,94371E-05 | 100,0 | 0,4 |
| 20 | TAPB DFTA COF 24 hours | 0,016356969 | 0,000211447 | 69,1 | 0,9 |
| 21 | PFBS 100 mg/L | 28,9579556 | 0,626835676 | 100,0 | 2,2 |
| 22 | TAPB DMTA COF 24 hours | 25,4260081 | 0,079294278 | 87,8 | 0,3 |
| 23 | PFBS 10 mg/L | 2,918136743 | 0,010359508 | 100,0 | 0,4 |
| 24 | TAPB DMTA COF 24 hours | 2,597267837 | 0,01107353 | 89,0 | 0,4 |
| 25 | PFBS 1 mg/L | 0,275851083 | 0,008121844 | 100,0 | 2,9 |
| 26 | TAPB DMTA COF 24 hours | 0,27475312 | 0,001013112 | 99,6 | 0,4 |
| 27 | PFBS 500 μg/L | 0,134318962 | 0,003426736 | 100,0 | 2,6 |
| 28 | TAPB DMTA COF 24 hours | 0,136217811 | 0,00220801 | 101,4 | 1,6 |
| 29 | PFBS 100 μg/L | 0,023664012 | 8,94371E-05 | 100,0 | 0,4 |
| 30 | TAPB DMTA COF 24 hours | 0,023652498 | 0,000175285 | 100,0 | 0,7 |

In addition to PFOA and GENX, the adsorption performance of the three COFs was also evaluated for GEN X and PFBS, as shown in Figure S12. The adsorption trends for these emerging PFAS compounds were generally consistent with those observed for PFOA and GENX. Among the three COFs, TAPB-DFTA COF consistently exhibited the highest adsorption capacity.

In kinetic experiments, TAPB-DFTA COF adsorbed 54.5 ± 2.9 % of GEN X and 45.8 ± 0.1 % of PFBS within 2 hours. Although substantial, these values were lower than the adsorption observed for PFOA and GENX under the same conditions. TAPB-TA COF demonstrated intermediate performance, removing 38.9 ± 1.8 % of GEN X and 28.2 ± 0.7 % of PFBS over the same period. TAPB-DMTA COF exhibited the lowest adsorption efficiency, capturing 31.6 ± 1.2 % of GEN X and 17.5 ± 1.9 % of PFBS after 2 hours of contact. A similar trend was observed in experiments conducted at higher PFAS concentrations with extended shaking times of 24 hours. For the highest concentration of GEN X (100 mg/L), TAPB-DFTA COF achieved 21.4 ± 1.3 % adsorption, followed by TAPB-TA COF at 15.2 ± 1.0 %, and TAPB-DMTA COF at 12.3 ± 2.4 %. In the case of 100 mg/L PFBS, TAPB-DFTA

COF adsorbed 4.9 ± 23.6 %, TAPB-TA COF removed 15.2 ± 0.4 %, and TAPB-DMTA COF achieved 12.2 ± 0.3 % adsorption.

Among the three materials, the fluorinated TAPB DFTA COF showed the highest adsorption of GenX and PFBS. However, adsorption efficiencies for these shorter-chain PFAS were generally lower than those observed for PFOA and PFOS.

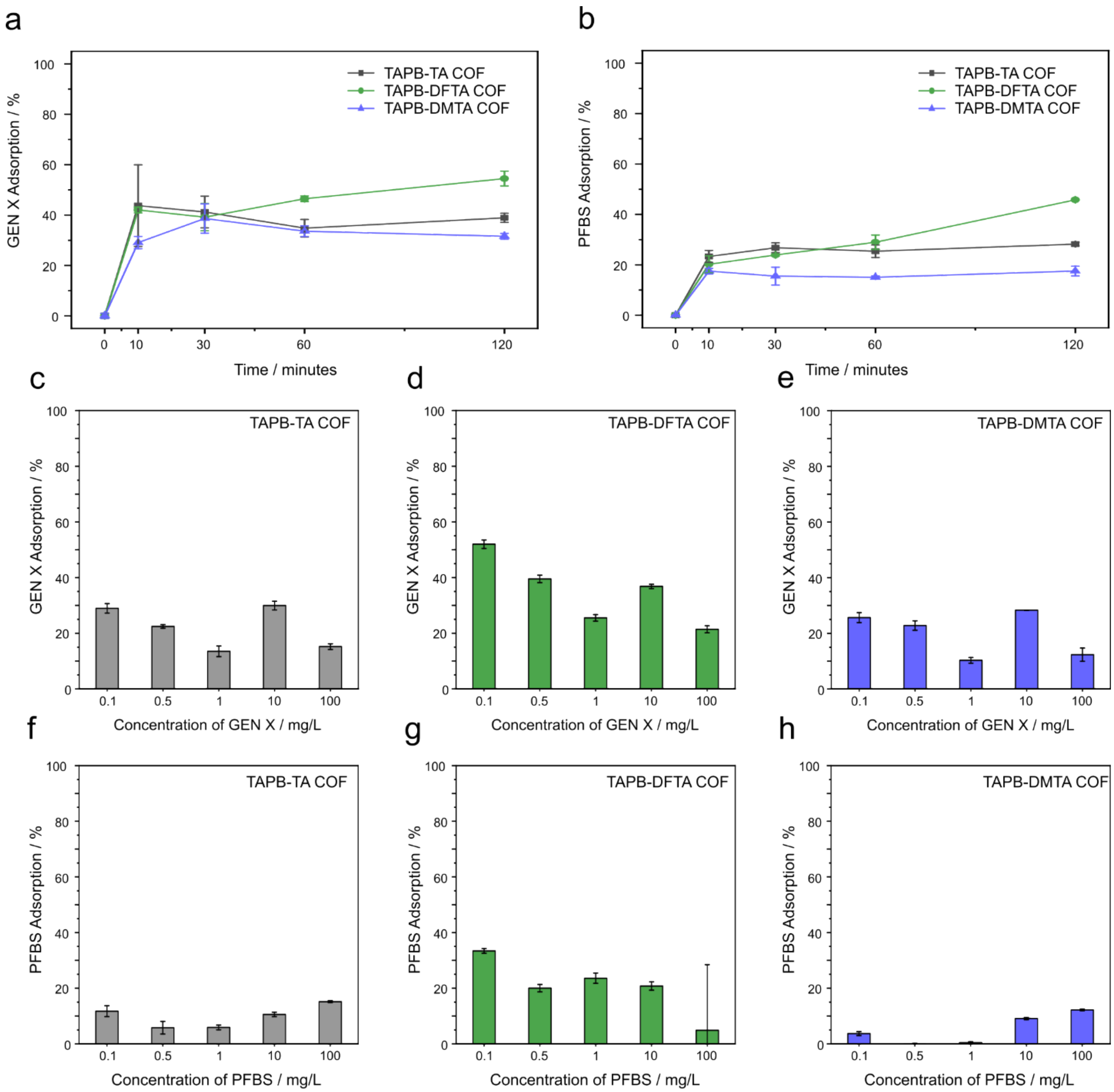


Figure S13: (a) Kinetic GEN X adsorption data of TAPB-TA COF, TAPB-DFTA COF and TAPB-DMTA COF. TAPB-DFTA COF was the best performer (b) Kinetic PFBS adsorption data of TAPB-TA COF, TAPB-DFTA COF and TAPB-DMTA COF. TAPB-DFTA

COF was the best performer here as well (c) Equilibrium GEN X adsorption data of TAPB-TA COF at different concentrations of GEN X (d) Equilibrium GEN X adsorption data of TAPB-DFTA COF at different concentrations of GEN X (e) Equilibrium GEN X adsorption data of TAPB-DMTA COF at different concentrations of GEN X (f) Equilibrium PFBS adsorption data of TAPB-TA COF at different concentrations of PFBS (g) Equilibrium PFBS adsorption data of TAPB-DFTA COF at different concentrations of PFBS (h) Equilibrium PFBS adsorption data of TAPB-DMTA COF at different concentrations of PFBS.

## S4.9 Correlation of SAXS analysis with PFAS adsorption

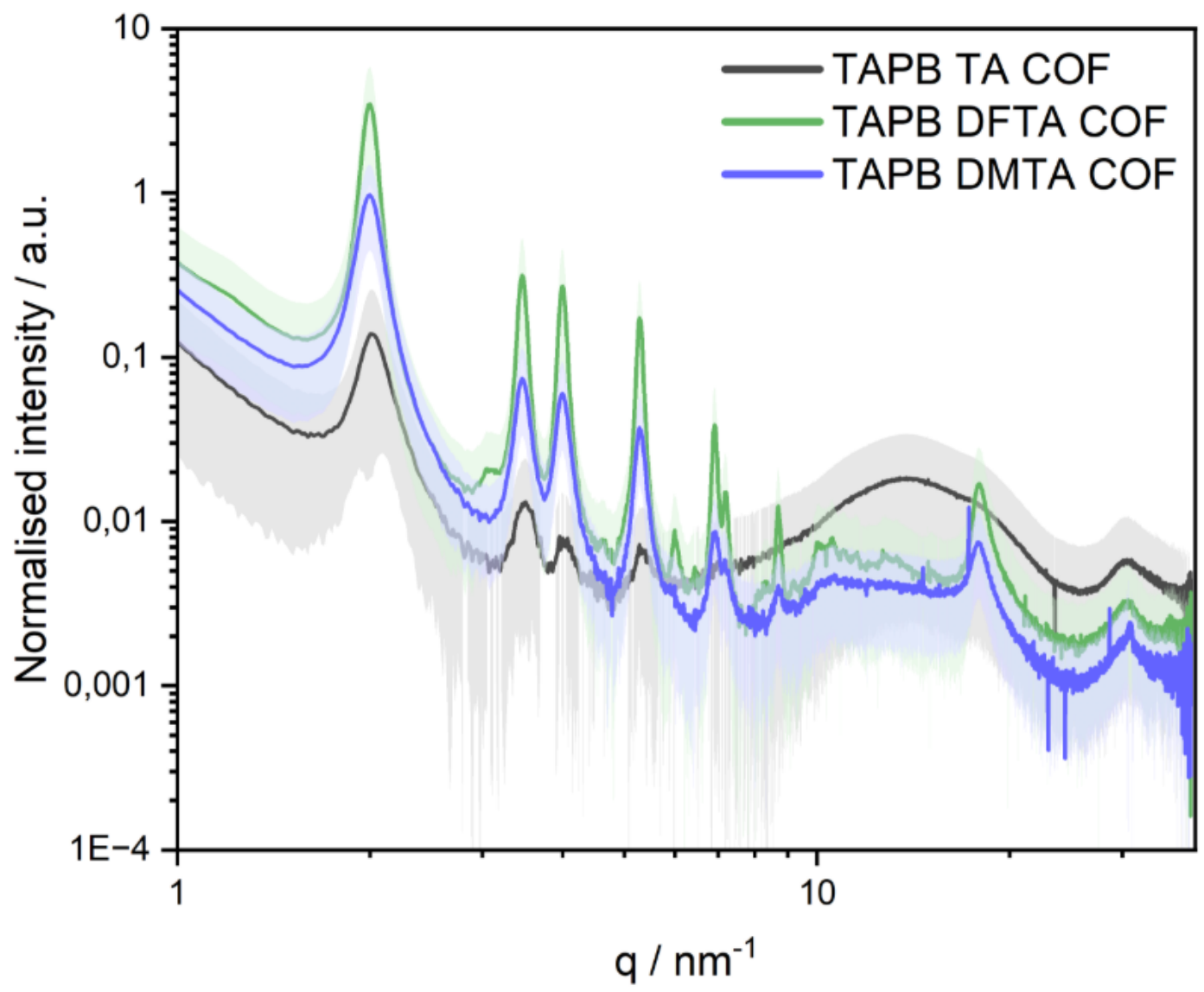


Figure S14: SAXS profiles of TAPB TA COF, TAPB DFTA COF and TAPB DMTA COF.

The SAXS measurements also revealed distinct differences in the degree of structural ordering among the three materials. The variations in their scattering profiles indicate that pore wall functionalization influenced the organization of the frameworks despite their isoreticular structures. The fluorinated TAPB DFTA COF exhibited the highest degree of crystallinity, followed by the methyl-functionalized TAPB DMTA COF while the unsubstituted TAPB TA COF showed the lowest degree of structural order. This is evidenced by the broader and less intense diffraction peaks at low q which originate from the periodic arrangement of the porous framework, compared with the corresponding reflections of the fluorinated and methyl-functionalized COFs. In addition, the SAXS profile of the TAPB TA COF is characterized by the appearance of form factor scattering at higher q together with the absence of distinct diffraction peaks in this region. This transition from structural diffraction to form factor scattering indicates a loss of long-range pore ordering rather than the complete absence of porosity.

Interestingly, these structural observations do not directly translate into the PFAS adsorption performance. As expected, the fluorinated COF exhibited the highest adsorption capacity, consistent with its superior surface area, pore characteristics and favorable fluorophilic interactions with PFAS molecules. However, the unsubstituted TAPB TA COF unexpectedly outperformed the methyl-functionalized TAPB DMTA material despite exhibiting both a lower BET surface area and reduced structural ordering according to SAXS. One possible explanation is that the lower degree of structural order is associated with the formation of smaller particles which may provide a greater accessible external surface for adsorption. Because the measured SAXS q range does not extend sufficiently into the Porod region, a quantitative determination of the specific external surface area cannot be derived from these measurements. Therefore, this interpretation should be regarded as a plausible hypothesis that is consistent with the observed morphology rather than a direct conclusion from the SAXS data. Additional evidence supporting this interpretation was obtained from ssNMR where the TAPB DMTA COF exhibited the coexistence of both AA and AB layer stacking. The presence of mixed stacking likely reduces pore accessibility and disrupts continuous transport pathways within the framework thereby limiting adsorption despite the comparatively higher BET surface area.

### S4.10 FT-IR analysis of COF before and after adsorption

Figure S15 presents the FT-IR spectra of the three COFs before and after GENX adsorption. In all cases, the characteristic imine stretching band is clearly observed, indicating that the imine linkages within the COF frameworks remain intact following the adsorption process. Notably, a slight broadening of the imine band is observed after GENX adsorption. Such broadening is commonly associated with interactions between the imine groups and adsorbed species, suggesting the involvement of the imine functionality in GENX binding.

This interaction is likely attributed to electrostatic attraction between the anionic sulfonate head group of GENX and partially protonated imine sites within the COF structure. In addition, new vibrational bands corresponding to the sulfonic acid group and the $CF_2$ moieties of GENX become evident after adsorption. The appearance of these characteristic GENX bands provides further confirmation of successful GENX uptake by the COFs.

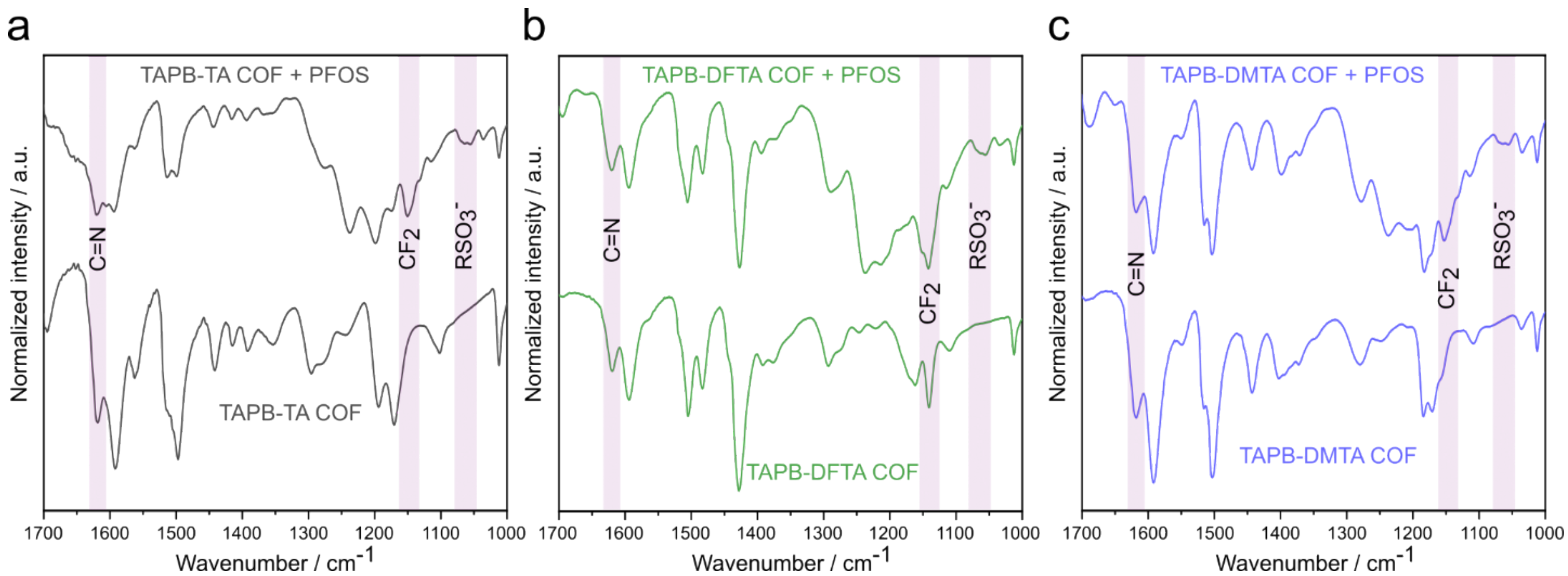


Figure S15: (a) FTIR spectra of TAPB-TA COF before and after GENX adsorption showing the presence of GENX in the framework. (b) FTIR spectra of TAPB-DFTA COF before and after GENX adsorption (c) FTIR spectra of TAPB-DMTA COF before and after GENX adsorption.
